# The History of Gene Hunting in Hereditary Spinocerebellar Degeneration: Lessons From the Past and Future Perspectives

**DOI:** 10.3389/fgene.2021.638730

**Published:** 2021-03-23

**Authors:** Ashraf Yahia, Giovanni Stevanin

**Affiliations:** ^1^Department of Biochemistry, Faculty of Medicine, University of Khartoum, Khartoum, Sudan; ^2^Department of Biochemistry, Faculty of Medicine, National University, Khartoum, Sudan; ^3^Institut du Cerveau, INSERM U1127, CNRS UMR7225, Sorbonne Université, Paris, France; ^4^Ecole Pratique des Hautes Etudes, EPHE, PSL Research University, Paris, France

**Keywords:** hereditary spastic paraplegia, hereditary cerebellar ataxia, spinocerebellar ataxia, spinocerebellar degeneration, gene discovery, diagnosis, neurogenetics

## Abstract

Hereditary spinocerebellar degeneration (SCD) encompasses an expanding list of rare diseases with a broad clinical and genetic heterogeneity, complicating their diagnosis and management in daily clinical practice. Correct diagnosis is a pillar for precision medicine, a branch of medicine that promises to flourish with the progressive improvements in studying the human genome. Discovering the genes causing novel Mendelian phenotypes contributes to precision medicine by diagnosing subsets of patients with previously undiagnosed conditions, guiding the management of these patients and their families, and enabling the discovery of more causes of Mendelian diseases. This new knowledge provides insight into the biological processes involved in health and disease, including the more common complex disorders. This review discusses the evolution of the clinical and genetic approaches used to diagnose hereditary SCD and the potential of new tools for future discoveries.

## Introduction

Hereditary forms of spastic paraplegia (SPG), cerebellar ataxia (CA), spastic ataxia, and spinocerebellar ataxia (SCA) are distinct clinical entities caused by related mechanisms and encompassing a continuum of phenotypes known as hereditary spinocerebellar degenerations (SCDs; Parodi et al., 2018). To date, they have been shown to be caused by pathogenic variants in more than 200 genes and loci inherited through X-linked, autosomal recessive, autosomal dominant, and mitochondrial patterns, and occasionally due to *de novo* events (Synofzik and Schüle, 2017; Parodi et al., 2018; Boutry et al., 2019; Manto et al., 2020; see Supplementary Table 1). SCDs are characterized clinically by cerebellar ataxia and/or spastic limbs, often complicated by other neurological or extra-neurological features (Parodi et al., 2018). The genetic and phenotypic heterogeneity of SCDs complicates their diagnosis (Elsayed et al., 2019). Moreover, the overlap between the clinical presentations of SCD and those of other genetic and non-genetic neurological conditions further complicates diagnosing these diseases (Elsayed et al., 2019). These diagnostic hurdles require the use of constantly evolving tools and approaches.

Spinocerebellar degenerations are estimated to affect 1:10,000 individuals worldwide, and thus fall within the category of rare diseases (Ruano et al., 2014; Nguengang Wakap et al., 2020). More than 70% of rare diseases are genetic, the majority of which affect the nervous system (Lee et al., 2020; Nguengang Wakap et al., 2020). From a biological perspective, Mendelian diseases can be viewed as natural “knockouts” or gains of function of a gene, a pathway, or a cellular process that can provide insight into complex biological systems and the more common complex diseases (Lee et al., 2020). From epidemiological and clinical perspectives, Mendelian diseases affect >2.5% of the human population (Nguengang Wakap et al., 2020), and therefore reaching a clinical diagnosis is valuable for the patients affected by these conditions and their families (Clark et al., 2018; Wright et al., 2018). The discovery of the cause of a Mendelian disease has, on some occasions, had a snowball effect and led to the discovery of more diseases (van der Knaap et al., 2002; Abou Jamra et al., 2011). Finding more causative genes helps to complete the puzzle and increases the probability of identifying biomarkers and therapeutic targets, hopefully of interest for multiple clinical-genetic entities.

**Figure 1:**
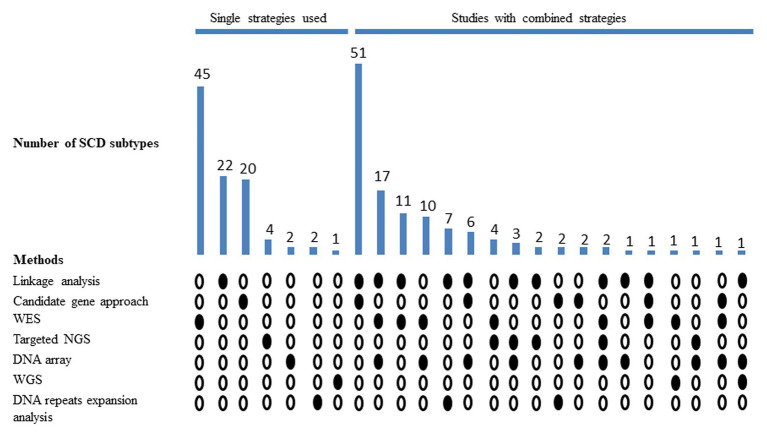
Methods used in diagnosing novel subtypes of hereditary spinocerebellar degeneration (SCD). Filled circles represent the methods used in identifying SCD subtypes, and the bar chart shows the number of SCD subtypes identified by each method or combination of methods. Empty circles represent the unused methods. SCD, hereditary spinocerebellar degeneration; WES, whole-exome sequencing; NGS, next-generation sequencing; and WGS, whole-genome sequencing.

The identification of SCD genes has passed several milestones, and multiple tools have been used in this quest (Figure 1; Supplementary Table 1). The evolution of the number of SCD entities has greatly benefited from improvements in terms of the tools used, their cost, the clinical phenotyping, and the availability of multiple samples in affected families. Several reviews have addressed the best practices to diagnose SCD subtypes at different periods (Harding, 1993; Fink, 1997; Brusse et al., 2007; De Silva et al., 2019; Shribman et al., 2019). Here, we review the evolution of the approaches used to identify novel SCD genes over time and discuss the possibility of combining some of these approaches with novel tools to increase the current SCD diagnostic rates and answer the current challenges in these diseases.

## Tools Used To Discover SCD Genes in the Past

### Linkage Analysis and Homozygosity Mapping

Whole genome linkage analyses, and homozygosity mapping in consanguineous cases, were the primary approaches for identifying genes and loci associated with Mendelian diseases (Lipner and Greenberg, 2018). These methods were developed in the 1980s thanks to improvements in detection of genetic markers and their assignment in chromosomal maps. These strategies were and are still, suitable for family-based approaches and realizable only in large pedigrees, although single nuclear sibships can be used in autosomal recessive consanguineous families (Lipner and Greenberg, 2018).

The linkage analysis concept relies on genetic variant co-segregation with nearby markers at the disease locus, as recombination is less likely to separate adjacent loci (Lipner and Greenberg, 2018). Microsatellite markers and array-based single nucleotide polymorphism (SNP) markers have been extensively used in SCD gene discovery. These markers replaced the restriction-fragment-length polymorphisms (RFLP) and the variable numbers of tandem repeats (VNTR) used in the 1980s. SNP markers are now commonly used either with DNA microarray or extracted from exome/genome sequencing data (Guergueltcheva et al., 2012; Novarino et al., 2014).

Among the 56% (123/220) of known SCD subtypes identified by approaches that involved linkage analysis, a good example of its diagnostic utility is the study by Novarino et al. (2014) that reported 15 novel causative genes in consanguineous families with various forms of SPG; they used SNP marker-based homozygosity to filter and prioritize the exome data. Indeed, most of the linkage-based SCD studies identified the responsible genes by coupling linkage analysis with candidate genes screening (51/220) or, more recently through biased (2/218) or unbiased next-generation sequencing (NGS; 31/220). For example, Guergueltcheva et al. (2012) pinpointed the mutation responsible for autosomal recessive spinocerebellar ataxia (SCAR) type 13 (SCAR13; OMIM # 614831) using linkage studies and mutational screening. There are still 22 SCD loci for which the causative gene has not been found and may have been missed if the causative variant is in non-coding regions, often remaining unexplored. Indeed, chromosomal location through linkage studies was critical for the identification of several of the nucleotide repeat expansion disorders such as the recently reported “cerebellar ataxia neuropathy and vestibular areflexia” syndrome (CANVAS) due to intronic repeat expansions in *RFC1* (Cortese et al., 2019).

The use of microsatellite or SNP markers is not confined to identifying disease loci but can extend to establishing the founder effects of disease-causing variants (Engert et al., 2000; Zivony-Elboum et al., 2012). This is clearly illustrated in studies performed to determine the ancestral origin of the pathological expansions in SCA3/Machado-Joseph disease (OMIM # 109150; Martins et al., 2007; Sharony et al., 2019).

### Repeat Expansion Detection Methods

Expansion of simple DNA sequence repeats causes more than 40 diseases, most with phenotypes affecting the central nervous system (Paulson, 2018; Rodriguez and Todd, 2019). They were first identified in a series of SCA loci in the 1990s following linkage studies and DNA/cDNA cloning strategies. These included the studies performed by Koide et al. (1994), David et al. (1997), and Koob et al. (1999) that directly screened patients with an autosomal dominant pattern of inheritance for repeat expansions and identified the genes responsible for dentatorubral-pallidoluysian atrophy (DRPLA; OMIM # 125370), SCA7 (OMIM # 164500), and SCA8 (OMIM # 608768). These discoveries benefited from the development of repeat expansion detection methods that used oligonucleotide probes containing triplet repeats and antibodies against polyglutamine tracks (Li et al., 1993; Trottier et al., 1995; Bahlo et al., 2018; Mitsuhashi and Matsumoto, 2020). The 1990s also witnessed the identification of the cause of Friedreich ataxia (OMIM # 229300), the most frequent autosomal recessive cerebellar ataxia commonly caused by intronic repeat expansions in the *FXN* gene (Campuzano et al., 1996).

There has been renewed interest in repeat expansion in recent years, with the development of diagnostic applications of PCR-based approaches (Cagnoli et al., 2018) and the improvement of algorithms for short-read sequence analysis. Recently, a combination of linkage analysis and short-read whole-genome sequencing (WGS) identified repeat expansions in *DAB1* and *RFC1* causing SCA37 (OMIM # 615945) and CANVAS (OMIM # 614575), respectively (Seixas et al., 2017; Cortese et al., 2019). In all likelihood, long-read sequencing methods will soon further increase the power of detecting repeat expansions (Bahlo et al., 2018; De Roeck et al., 2019; Mitsuhashi and Matsumoto, 2020). Up to now, methods for detecting DNA repeat expansions were applied in discovering 5% (11/220) of the SCD subtypes, mostly (9/11) when coupled with whole genome linkage analysis.

### Candidate Gene Approaches

Prioritizing genes is a complementary but crucial step in all the approaches taken to identify SCD genes. This prioritization usually depends on the gene expression, the gene’s function or its product’s function, and the phenotype of patients and/or animal models. Interestingly, a straightforward candidate gene approach was the main methodology in identifying 9.1% (20/220) of SCD subtypes.

There are multiple mouse models of neurodegeneration, particularly with cerebellar features, and they can be very informative in guiding candidate gene selection. Escayg et al. (2000) screened patients with neurological phenotypes for mutations in the *CACNB4* gene after they had identified bi-allelic mutations in *CACNB4* in a mouse model presenting with ataxia and epilepsy, and they identified *CACNB4* as the cause of type 5 episodic ataxia (EA5; OMIM # 613855; Burgess et al., 1997). Loss of function variants in the *NHE1* gene coding the sodium-hydrogen exchanger 1 (NHE1) are associated with Lichtenstein-Knorr syndrome (OMIM# 616291), a cerebellar syndrome complicated by sensorineural hearing loss (Guissart et al., 2015). Interestingly, the Lichtenstein-Knorr syndrome’s phenotype is partially observed in the mouse knockout for *NHE1* and for *CHP1* as well, coding an NHE1 interactor protein (Bell et al., 1999; Liu et al., 2013). These observations prompted Mendoza-Ferreira et al. (2018) to prioritize a frame-shift variant in *CHP1* observed on whole-exome sequencing (WES) data of Moroccan patients with complex cerebellar ataxia and intellectual disability, the first cases of spastic ataxia type 9 (OMIM # 618438). Human genetics can also benefit animal genetics. This is shown by the identification of a *KIFC* variant in Charolais cattle developing a spastic ataxia phenotype by prioritization of the WGS data on this gene after it was involved in a similar human disorder (Duchesne et al., 2018).

The clinical and biological features of the patients have also helped to point to specific candidate genes. The patients’ clinical presentation and enzymatic assays significant for ubiquinone deficiency led to the implication of pathogenic mutations in *ADCK3* in SCAR type 9 (SCAR9; OMIM # 612016; Mollet et al., 2008). The patients’ phenotype and hormonal assays implying thyroid hormone resistance led to the implication of *SLC16A2* mutations in X-linked SPG22 (OMIM # 300523; Friesema et al., 2004). Similarly, the clinical phenotype led to the discovery of some SCD subtypes caused by mitochondrial alterations (Holt et al., 1990; Winterthun et al., 2005; Sarzi et al., 2007).

Many attempts to identify the SCD genes’ interactors have been performed using yeast two-hybrid screening (Lim et al., 2006) and *in silico* networks (Novarino et al., 2014). The latter study identified three causative genes in HSP families using an HSP *in silico* functional network (Novarino et al., 2014). Based on this “HSP network,” the authors selected three candidate genes and tested them by direct sequencing in a large series of HSP cases, a strategy that led to *REEP2*, *MAG*, and *BICD2* being identified as novel HSP genes (Novarino et al., 2014). Along the same lines, Słabicki et al. (2010) adopted another approach in identifying the gene responsible for SPG48; they first performed RNAi genome-wide screening in HeLa cells for genes involved in DNA double-strand breaks and identified the *AP5Z1* gene among the modifier genes. *AP5Z1* was shown to interact with spastascin and spastizin, the proteins involved in SPG11 and SPG15, respectively. Słabicki et al. (2010) then sequenced the *AP5Z1* gene in 166 patients with unexplained HSP and identified the first SPG48 case. These studies demonstrated the usefulness of knowing the interactors of the SCD proteins as they are likely the source of potential variants in patients.

Knowledge of the physiopathology at the cell level can also guide the candidate gene analysis. Jen et al. (2005) screened a cohort of patients with unexplained episodic ataxia and hemiplegic migraine for mutations in *SLC1A3*, which encodes a glutamate transporter essential for removing glutamate from the synaptic cleft. Glutamate is the most abundant amino acid neurotransmitter in the central nervous system. In their study, Jen et al. (2005) identified a heterozygous mutation in *SLC1A3* in a patient with episodic ataxia called later episodic ataxia (EA) type 6 (EA6; OMIM # 612656).

Other studies have implicated genes known for their association with specific neurological phenotypes in developing new SCD forms based on phenotypic similarities between the old and the new phenotypes. These include studies that implicated *SCN2A* in EA9 (OMIM # 618924; Liao et al., 2010), *GJC2* in SPG44 (OMIM# 613206; Orthmann-Murphy et al., 2009), *PGN* in the autosomal dominant SPG7 (Sánchez-Ferrero et al., 2013), *FMR1* in fragile X tremor/ataxia syndrome (FXTAS, OMIM # 300623; Hagerman et al., 2001), and *DARS2* in hereditary SPG (Lan et al., 2017).

### DNA Microarrays

DNA microarrays are used in clinical settings to investigate copy number variations (CNVs) and can also help determine SNP genotypes for linkage analysis or homozygosity mapping strategies (Levy and Burnside, 2019). DNA microarrays used for CNV detection are the first-tier tests for diagnosing neurodevelopmental disorders, disease entities that include intellectual disabilities, developmental delay disorders, autism spectrum disorders, and disorders with multiple congenital anomalies (Jang et al., 2019). However, a recent meta-analysis suggested that WES outperforms microarrays in diagnosing neurodevelopmental disorders (Srivastava et al., 2019). Furthermore, in a recent study performed over a 10-year period, Ciaccio et al. (2020) discouraged the use of chromosomal microarrays as the first-tier test for diagnosing neurodevelopmental disorders with ataxia.

DNA arrays were the sole genetic screening tool used in discovering two SCD genes. Moreno-De-Luca et al. (2011) used a custom-designed oligonucleotide array in discovering *AP4E1*, the gene responsible for SPG51 (OMIM# 613744). Moreno-De-Luca et al. (2011) used a genome-wide microarray in their study to identify a region of interest, and then further analyzed the identified region with a higher-resolution microarray. In another study, Utine et al. (2013) used an SNP array in discovering the gene causing SCAR18 (OMIM # 616204). Guided by array-based homozygosity mapping, Utine et al. (2013) identified deletions in the third and fourth exons of *GRID2* in three Turkish siblings. They confirmed the absence of these exons in the patients using real-time PCR (RT-PCR).

Coupling arrays to other genetic screening tools has led to the identification of 20% (44/220) of all currently known SCD genes. More than half of the genes thus identified (27/44) were discovered using approaches that involved microarrays (mainly for linkage studies) and WES.

### Next-Generation Sequencing Approaches

The advent of NGS has revolutionized the field of medical genetics as it enabled parallel sequencing of massive targets in a short duration of time and for a plummeting cost (Boycott et al., 2017; Mazzarotto et al., 2020). NGS is classified according to the targeted sequences. The targeted sequence in WGS is the whole genome, in WES it is the coding part of the genome, and in targeted NGS (TS) it is a custom-ordered set of genes and sequences (Gao and Smith, 2020). NGS is also classified as long-read sequencing and short-read sequencing according to the length of the sequenced fragments, also known as the sequence reads. The length of the sequence read in short-read sequencing is ~75–300 base-pairs, while in long-read sequencing; it is equal to the length of the template sequence, at least in theory (Caspar et al., 2018).

**Figure 2:**
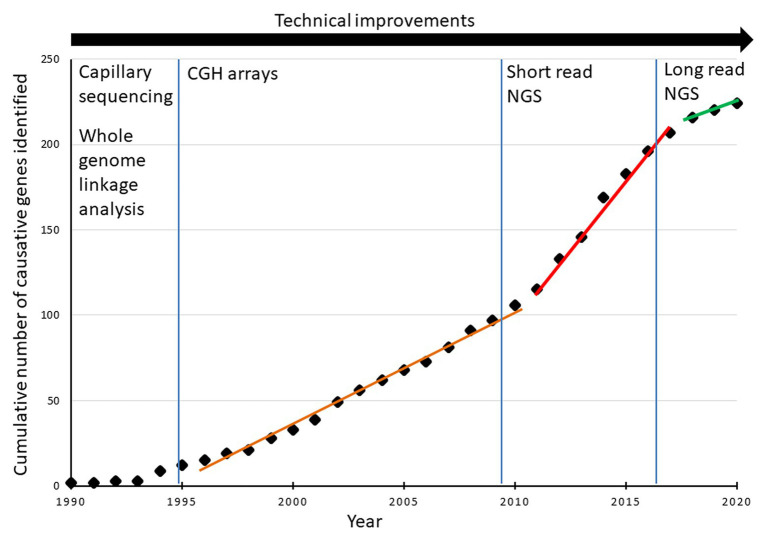
Cumulative evolution of the number of subtypes of hereditary SCD identified per year.

Next-generation sequencing is extensively and routinely used in diagnosing Mendelian diseases, including those with neurological phenotypes (Bhatia et al., 2020; Rouleau et al., 2020). To date, 47.3% (104/220) of SCD genes have been discovered using NGS-based approaches, and the slope of the curve in SCD gene discovery drastically changed from 2009, when this technique was made available (Figure 2).

#### Targeted NGS

Targeted NGS is the most commonly used NGS tool in clinical practice (Gao and Smith, 2020). In the clinical context, TS investigates the roles of a predetermined set of genes in a clinical phenotype (Gao and Smith, 2020). In the setting of research and gene discovery, researchers use TS to focus on specific regions and sequences of interest determined by the clinical data or other diagnostic modalities.

Targeted NGS was involved in the discovery of 7.3% (16/220) of known SCD subtypes, mostly (73%) when coupled with other diagnostic modalities. TS was the primary genetic test used in four studies that identified four new SCD subtypes. In the first, Zanni et al. (2012) sequenced all the exons in the X chromosome in a family with X-linked cerebellar ataxia and identified a missense mutation in the *ATP2B3* gene. Pathogenic mutations in *ATP2B3* cause X-linked SCA type 1 (SCAX1; OMIM # 302500; Zanni et al., 2012). In the other three studies, TS identified mutations in *SPTBN2*, *KIF1A*, and *TUBB4A* as the causes of SCAR14 (OMIM # 615386), the autosomal dominant form of SPG30 (OMIM # 610357), and *TUBB4A*-associated SPG, respectively (Lise et al., 2012; Ylikallio et al., 2015; Sagnelli et al., 2016). Pathogenic mutations in *SPTBN2*, *KIF1A*, and *TUBB4A* were already known to cause SCA5 (OMIM # 600224), autosomal recessive SPG30 (OMIM # 610357), and both autosomal dominant type 4 dystonia (OMIM # 128101) and type 6 hypomyelination leukodystrophy (OMIM # 612438), respectively (Ikeda et al., 2006; Erlich et al., 2011; Hersheson et al., 2013; Simons et al., 2013). Thus, the three subsequent studies added SCD to the clinical phenotypes associated with genes previously known to cause diseases other than SCD, a situation becoming more frequent in recent years than real gene identification in SCD as shown by the change in the slope of the curve in Figure 2 since 2017.

Targeted NGS is mainly used in genetic diagnosis in clinical practice as it allows focused analysis, thereby limiting incidental findings (Elsayed et al., 2016; Morais et al., 2017). In addition, given that it is optimized for a specific set of genes, sequence capture is usually homogeneous, allowing CNV detection through coverage analysis (Moreno-Cabrera et al., 2020).

#### Whole-Exome Sequencing

Whole-exome sequencing covers ~1–2% of the human genome and is extensively used for diagnosing Mendelian diseases (Hansen et al., 2020; Rouleau et al., 2020). The 3' and 5' untranslated regions (UTRs) are also included in most WES kits, at a variable level according to the company; however, variants in these regions are often difficult to analyze and interpret (Devanna et al., 2018). To date, mutations in ~20% of the protein-coding genes are known associated with a disease trait (Posey, 2019).

There are two views on whether TS or WES is the best first-tier test for diagnosing patients with SCDs (Galatolo et al., 2018; Shribman et al., 2019). TS has a lower cost, higher depth, lower analysis time, and fewer incidental findings (Gao and Smith, 2020; Mazzarotto et al., 2020; Platt et al., 2020). WES has higher coverage and outperforms TS in discovering new Mendelian disease-causing genes (Gao and Smith, 2020; Mazzarotto et al., 2020; Platt et al., 2020). WES was the only genetic screening tool used in discovering 20.5% (45/220) of known SCD subtypes and an additional 21.4% (47/220) when coupled with other technics. Focused exome sequencing (FES) is a WES-based approach that possesses some of the advantages of TS and WES (Pengelly et al., 2020). FES has higher coverage than TS, as it currently targets ~5,000 disease-associated genes, ~20% of the sequences targeted by WES (Pengelly et al., 2020). FES has greater depth and produces fewer incidental findings compared to WES but has a lower potential for discovering new SCD genes (Pengelly et al., 2020).

The diagnostic yield of WES is higher in familial cases compared to sporadic cases (Coutelier et al., 2018; Ngo et al., 2020). However, this generalization has some exceptions. For example, Fogel et al. (2014) reported a success rate of 73% (22/30) in diagnosing patients with sporadic cerebellar ataxia. Reanalysis of the undiagnosed exomes and coupling WES with other genetic approaches, such as mRNA sequencing and CNV detection, also increase WES’s diagnostic yield (Deelen et al., 2019; Jalkh et al., 2019; Fung et al., 2020; Matalonga et al., 2020). CNVs are common in some SCD subtypes, e.g., SPG4 (OMIM # 182601) and SCA15 (OMIM # 606658; Depienne et al., 2007; Hsiao et al., 2017). They can be looked for in short-reads sequencing data, but with variable rates according to the used algorithms and the library preparation, making comparative genomic hybridization (CGH) arrays or multiplex-ligation-probe-amplification (MLPA) favored to detect CNV. One study reported a rise of 7% in the diagnostic success rate upon reanalyzing the WES data of a cohort of SCD patients (Ngo et al., 2020). Recent reports advocate for a periodic reanalysis of WES data (Jalkh et al., 2019; Liu et al., 2019; Fung et al., 2020; Ngo et al., 2020). The 5-year cumulative increase in the diagnostic success rate upon WES data reanalysis ranges from 12 to 22% (Liu et al., 2019).

#### Whole-Genome Sequencing

Whole-genome sequencing is the most comprehensive tool to study the genome (Gao and Smith, 2020). A significant advantage of WGS is its ability to interrogate intergenic and intronic regions that constitute most of the human genome. This ability is a double-edged sword as the interpretation of intergenic and intronic variants is hard compared to the interpretation of exonic variants (Gao and Smith, 2020). For that reason, most of the time, WGS is interrogated for exonic sequences first, and often, limited to them (Rexach et al., 2019). WGS has a lower but more uniform coverage compared to WES (Posey, 2019). This uniformity in coverage of exonic sequences has resulted in better detection of GC-rich regions, e.g., at the first exons, and CNVs, compared to WES (Posey, 2019; Rexach et al., 2019).

To date, WGS has been involved in the discovery of three SCD genes. Melo et al. (2015) identified a homozygous 216-bp deletion in the non-coding sequence upstream to the *KLC2* gene in patients with SPG, optic atrophy, and neuropathy (SPOAN). Melo et al. (2015) identified this 216-bp deletion in screening a series of 275 patients (273 from Brazil and two from Egypt) with SPOAN and validated its pathogenicity in a zebrafish model. The candidate region containing *KLC2* was refined using linkage analysis and WES (Macedo-Souza et al., 2009; Melo et al., 2015). Similarly, Cortese et al. (2019) identified bi-allelic intronic expansions in *RFC1* in 56 patients from the United Kingdom, Italy, and Brazil with the CANVAS phenotype using a WGS-based approach focused to the linked-chromosomal region. In another study, Wagner et al. (2019) using WGS, identified a biallelic splice-site variant in the *RNF170* gene in a family with early-onset HSP complicated with peripheral neuropathy. Wagner et al. (2019) corroborated their finding by identifying additional patients with pathogenic *RNF170* variants on the GENESIS platform^1^ of shared sequencing data (Gonzalez et al., 2015).

Studies that follow the WGS-based approach to identify novel genes generally use other techniques, such as whole-genome linkage analysis, to focus the search for candidate variants. Indeed, the bottleneck of the WGS-based approach is the large number of variants that can be identified. Multiomics integration will ameliorate the interpretation of the functional and regulatory effects of variants identified by NGS, especially WGS (Agrawal et al., 2014; Posey, 2019). Furthermore, long-read WGS is probably a promising new tool for diagnosing genetic diseases for the near future with great hope on detection of nucleotide repeats as well (Posey, 2019; Wenger et al., 2019).

## Discussion and Future Directions

### The Need for Combined Approaches

Genome-wide approaches have proved the most successful in identifying new SCD subtypes, having identified 86.4% (190/220) of the known SCD subtypes. These approaches have made use of linkage analysis, WES, WGS, and DNA microarrays. Linkage analysis with candidate gene screening dominated the discovery of SCD genes in the past, often thanks to prior knowledge of these genes’ functions and expression profiles, their products, the pathways they serve, or pertinent animal models. WES is now dominating SCD gene discovery and we can expect that WGS will dominate the discovery of the genes causing Mendelian diseases in the future, probably within integrated multi-omics strategies to reduce the number of candidate variants (Posey, 2019; Kerr et al., 2020).

**Figure 3:**
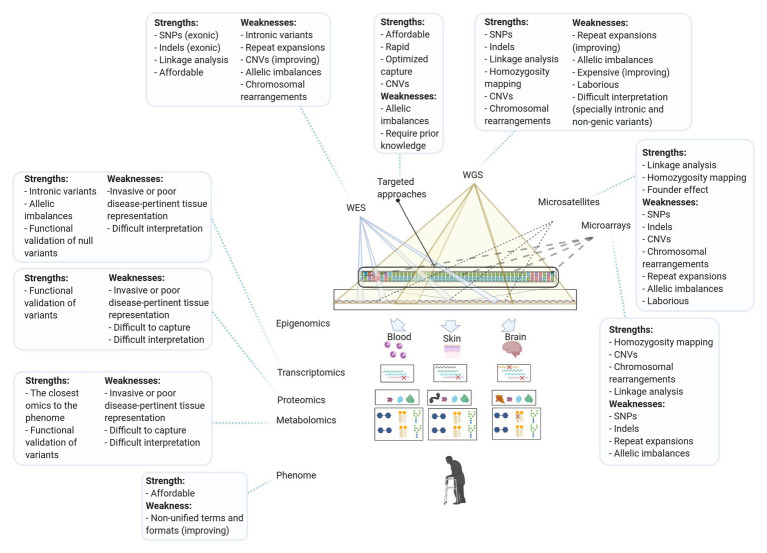
The strengths and weaknesses of the methods used in diagnosing subtypes of hereditary SCD. WES, whole-exome sequencing; WGS, whole-genome sequencing; Targeted approaches, targeted next-generation sequencing, candidate gene approaches, and repeats expansion detection methods; SNPs, single nucleotide polymorphisms; and CNVs, copy number variations.

Indeed, approaches that utilized more than one genetic screening tool identified 55.9% (123/220) of SCD subtypes compared to the 43.2% (95/220) identified by single tools. Historically, the most successful approaches in identifying SCD genes were those involving linkage analysis and exome sequencing. However, the use of NGS to diagnose rare diseases has been accompanied by a shift away from studying single large multiplex pedigrees with a restricted phenotype toward studying phenotypically diverse multiple small pedigrees and sporadic cases as well (Wright et al., 2018; Claussnitzer et al., 2020). In our opinion, WES and SNP arrays are the best combination to accelerate the discovery of SCD genes, since SNP arrays can be used in detecting CNVs, chromosomal aberrations, and linkage/homozygosity regions (Levy and Burnside, 2019). SNP arrays are also the most widely used tools for studying complex traits by genome-wide association studies (Tam et al., 2019). WGS could achieve all these goals, in an even better way, and eliminate the need to couple WES and SNP arrays (Figure 3). However, at the present time, the use of WGS on a broad scale is prohibited by its relatively high costs and the technical difficulties in storing, analyzing, and sharing WGS data (Suwinski et al., 2019; Tam et al., 2019).

### Emerging Approaches

Several tools and developments in the genome-wide approaches have emerged to have inherent potentials for augmenting the discovery of new Mendelian diseases. These advancements include, but not limited to, the optical mapping methods for detecting genomic rearrangements (e.g., Bionano optical mapping), long-read genomic sequencing valuable for detecting copy number variants and nucleotide expansions (e.g., Oxford Nanopore or PacBio technologies), and mosaicism detection tools (e.g., MosaicForecast; Lam et al., 2012; Jain et al., 2018; Dou et al., 2020). Structural rearrangements, CNVs, sequence expansions, and somatic mutations account for a significant proportion of the missing heritability in Mendelian diseases (Maroilley and Tarailo-Graovac, 2019).

The comprehensiveness of WES and WGS should not lead us to underestimate the role of proper clinical phenotyping in diagnosing genetic diseases (Pena et al., 2018). Tools that incorporate phenotypic data in NGS analysis pipelines are under development and have been used with success (Thuriot et al., 2018; Zhao et al., 2020). These include tools that empower the clinicians to have a central role in prioritizing variants, e.g., tools that apply gene-pertinence metrics (Segal et al., 2020). Proper clinical phenotyping is of value to increase the diagnostic rate of rare diseases (Bhatia et al., 2020). The high degree of overlap between various neurodegenerative disorders, including SCD, calls for better documentation, and follow-up for patients (Klebe et al., 2015; Parodi et al., 2018). This is clearly seen by the constant identification of more genes involved in other diseases and found mutated in SCD as well. The reverse is also true and illustrates the clinical overlap with various diseases. International registries, such as SCA Global and ARCA Global^2^ and SPATAX,^3^ have been launched to meet the need for better documentation of SCD cases. A prerequisite for international patient registries, besides appropriate research ethics policies, is the development of unified common disability scales to facilitate analysis and comparison (Trouillas et al., 1997; Lin et al., 2020). Many disability scales have been developed for SCD, the most commonly used scales being the scale for the assessment and rating of ataxia (SARA) and the International Cooperative Ataxia Rating Scale (ICARS; Perez-Lloret et al., 2020). Disability scales do not only measure disability; unlike what the name implies, but they can also measure the response to therapeutic interventions including attempts to preserve motor capacities of patients through alternative treatment options, e.g., dance, video game-based coordinative training (exergames), etc. (Ilg et al., 2012).

On the other hand, clinical phenotypes will, in many cases, evolve over time, and this needs to be considered when analyzing NGS data (Liu et al., 2019; Bhatia et al., 2020). Artificial intelligence tools have recently been employed in phenotyping, known as next-generation phenotyping, with good results, especially in neurological diseases (Elmas and Gogus, 2020; Pode-Shakked et al., 2020).

Experimental forward genetics has identified a few SCD genes. Forward genetics investigates the genetic basis of a particular phenotype, in contrast to reverse genetics, which investigates the phenotypic consequences of altering a particular gene (Moresco et al., 2013). The current development in forward genetics and its equipment with NGS tools could further enhance the application of forward genetics for discovering new genes implicated in Mendelian diseases (Singh, 2020). Furthermore, gene essentiality screens on model organisms and cell lines of different origins are gaining momentum and are progressively integrated into gene-prioritization steps in NGS analysis pipelines (Cacheiro et al., 2020).

### Remaining Challenges

There is still a pressing need for biomarkers to complement the advances in SCD genomics and phenomics. The role of biomarkers is not limited to diagnosis, as biomarkers can objectively measure disease evolution, prognosis, and response to therapy (Gülbakan et al., 2016). Currently, the means for validating the biological impact of candidate pathogenic SCD variants in the clinical setting are not available except for a few SCD subtypes, e.g., SPG5A caused by mutations in the *CYP7B1* gene (27-OH cholesterol), SPG9A (OMIM # 601162), and SPG9B (OMIM # 616586) caused by mutations in the *ALDH18A1* gene (amino-acid levels in blood), and ataxia with isolated vitamin E deficiency (OMIM # 277460) caused by mutations in the *TTPA* gene (Ouahchi et al., 1995; Coutelier et al., 2015; Marelli et al., 2018).

The rapidly evolving field of metabolomics is expected to contribute to the SCD biomarkers list (Wishart, 2016; Yang et al., 2019). Metabolomics is analyzing the body metabolites in a comprehensive, yet systematic approach (Gülbakan et al., 2016). Metabolic disturbances are core pathological mechanisms in many SCD subtypes (Blackstone, 2018; Darios et al., 2020). Consequently, metabolomics will not only provide biomarkers but also will shed light on the SCD biochemistry and provide an objective means for classifying SCD subtypes (Wishart, 2016; del Mar Amador et al., 2018; Yang et al., 2019). It can also unravel the overlapping phenotypes caused by mutations in a single gene, a pattern common in neurological diseases, including SCD (Esterhuizen et al., 2021). Developing an objective SCD nosology is a prerequisite for advancing SCD research and therapeutics (Elsayed et al., 2019).

Metabolomics is the closest omics layer to the phenotype and could have central roles in validating the newly discovered Mendelian genes (Feussner and Polle, 2015; Graham et al., 2018; Almontashiri et al., 2020). Metabolomics studies are conducted using two main approaches: a hypothesis-directed approach that studies a predetermined set of metabolites (targeted metabolomics) and a hypothesis-free comprehensive approach that aims to analyze the whole set of metabolites within a system (untargeted or global metabolomics; Almontashiri et al., 2020; Chen et al., 2020). Global metabolomics is gaining momentum over targeted approaches in clinical settings (Almontashiri et al., 2020). However, neither approach is suitable yet for the *ab initio* discovery of new genes; rather, global metabolomics can be integrated with exome and genome sequencing to enhance new genes discovery (Graham et al., 2018; Almontashiri et al., 2020).

Multiple hurdles are facing the application of metabolomics in Mendelian genes discovery. Firstly, the human metabolome is made of thousands of compounds influenced by the genome but also by a plethora of non-genomic determinants, including the microbiome, lifestyle, environment, and others (Bar et al., 2020). Secondly, a proportion of the human metabolome is not identified yet and there is no single tool currently capable of capturing the whole metabolome (Graham et al., 2018). Furthermore, there is no congruence between the concentration of certain metabolites in the blood and the brain (Yang et al., 2019). Lastly, the limited number of patients with rare diseases, particularly those in whom new phenotypes are discovered, complicates the analysis of metabolomics data and the differentiation between genuine signals and background noise (Graham et al., 2018; Yang et al., 2019). However, recently, some promising solutions have emerged to overcome those hurdles.

Multiple repositories were launched to enhance metabolomics data sharing, and, in parallel, tools that integrate the data from different repositories were recently developed (Krassowski et al., 2020; Palermo et al., 2020). Examples for such repositories include MetaboLights, GNPS-MASSIVE, and Metabolomics Workbench (Haug et al., 2013; Sud et al., 2016; Wang et al., 2016). Animal models can be used to alleviate the need for human tissues to study the metabolome (Kilk, 2019). Even more promising are the human organoids, which are more representative substitutes for the human brain tissues (Kim et al., 2020). To a large extent, organoids can also solve the scarcity of patients with rare diseases (Kim et al., 2020).

Other omics layers, particularly the transcriptome and the proteome, can be exploited in the quest for identifying new SCD genes with the limitation of the access to the brain tissue in most cases. A substantial proportion of the undiagnosed Mendelian diseases are caused by variants impacting RNA expression and/or regulation, including deep intronic variants (Gonorazky et al., 2019). Transcriptome sequencing (RNA-seq) is deemed to enhance identifying Mendelian variants by ~10–30% (Kremer et al., 2017; Gonorazky et al., 2019). Allelic imbalances in transcriptomics data can identify imprinting, uni-parental disomy, and X chromosome inactivation (Gonorazky et al., 2019; Yépez et al., 2021). However, the tissue transcriptome does not necessarily reflect the proteome, metabolome, and phenome (Wang et al., 2019). In the abnormal tissues, the discrepancy between the transcriptome and the proteome can be more evident depending on the underlying disease’s pathophysiology and the type of the disease-causing variants (Roos et al., 2018). Although the proteome is closer to the phenome than the transcriptome, it is more easier to recover data by RNA-seq than proteome-capturing approaches due to the higher depth of RNA-seq and the much wider proteins’ dynamic range of expression (Feussner and Polle, 2015; Wang et al., 2019). Moreover, ~13% of the human proteome is still missing and not captured (Omenn et al., 2017; Wang et al., 2019).

A holistic approach that integrates data obtained from multiple omics layers (multi-omics approach) has a higher biological reliability and can substantially enhance new Mendelian genes’ discovery than the single-omics approaches (Labory et al., 2020). The multi-omics have been successfully used in many diseases, including neurological diseases, and is thus a promising approach to enhance the discovery of new SCD genes (Crowther et al., 2018; Kerr et al., 2020; Labory et al., 2020).

In the current big data era, tools that integrate the data generated at different biological scales, namely multi-omic tools, are rapidly emerging (Anderson et al., 2019; Kerr et al., 2020; Subramanian et al., 2020). This promotes collaborative studies. In some instances, establishing associations between variants, especially non-exonic variants, and diseases, required a large number of patients and controls. Involving patients from different genetic backgrounds has aided the refinement of the loci associated with some SCD genes. An increasing number of consortia and collaborative platforms have been launched to enhance the discovery of new genes by enabling the sharing of sequence data, e.g., GENESIS platform; variants and phenotypes, e.g., GeneMatcher; and connecting geneticists and biologists to enhance validating new candidate genes, e.g., the Canadian Rare Diseases Models and Mechanisms Network (Gonzalez et al., 2015; Sobreira et al., 2015; Boycott et al., 2020). Larger-scale collaborations are essential to explore a very challenging aspect of all neurogenetic conditions, the clinical variability. Modifier genes can explain part of the phenotypic variability commonly seen between patients, as shown in autosomal dominant SCAs (Du Montcel et al., 2014).

Sporadic cases remain a challenge in these diseases. They are often the most frequent in clinical practice and their analysis possibility is limited. Collaborations, trio analysis, and multi-omic approaches can assist in solving them. In our experience, 10% of sporadic cases can be explained by *de novo* variants or inherited variants due to incomplete penetrance, adoption, family censure, etc. We expect multigenic alterations or somatic variants to explain as well part of the missing heritability in some of the patients with sporadic forms.

Finally, one of the bottlenecks in diagnosing SCD is the functional validation of the variants of unknown significance (VUS). Three-dimensional modeling and functional enzymatic assays can be precious, as shown for variants in the *CYP2U1* gene, the gene associated with SPG56 (OMIM # 615030; Durand et al., 2018). Small animal models such as *C. elegans* and zebrafish can also be used (Martin et al., 2013; Novarino et al., 2014; Chiba et al., 2019). Moreover, organoids emerged as promising models to establish pathogenicity and circumvent the difficulty in obtaining brain tissues (Kim et al., 2020). Without such systematic studies, we can reasonably think that a significant part of variants are not really causative which may limit the finding of phenotype-genotype correlations. In that respect, the report of second mutated families is required for multiple clinico genetic entities, including SPG59, 60, 65, 67, 68, 69, 70, and 71, found in single families yet (Novarino et al., 2014).

## Conclusion

In conclusion, the toolbox for diagnosing Mendelian diseases is continuously expanding, including multiple “out of the box” approaches. These valuable utensils and approaches (Figure 3) have solved and deemed to continue solving some of the Mendelian diseases diagnostic odysseys. This review highlighted some of the approaches successfully used to discover SCD genes and shed light on some of the promising resources and approaches expected to play central roles in diagnosing SCD soon. Discovering novel genes associated with SCD is moving toward integrated multi-omic research using innovative technologies. We hypothesize that many neurogenetic entities will join the spectrum of SCD, necessitating newer and more comprehensive nosologies at the SCD, and likely at the neurogenetic level as well.

## Author Contributions

AY and GS designed, wrote, and critically revised this review article. All authors contributed to the article and approved the submitted version.

### Conflict of Interest

The authors declare that the research was conducted in the absence of any commercial or financial relationships that could be construed as a potential conflict of interest.

## Footnotes

^1^https://www.tgp-foundation.org/genesis-log-in

^2^http://ataxia-global-initiatives.net/

^3^https://spatax.wordpress.com/
