# Supplementary material for: The History of Gene Hunting in Hereditary Spinocerebellar Degeneration: Lessons From the Past and Future Perspectives
Source: Front Genet. 2021 Mar 23;12:638730. doi: 10.3389/fgene.2021.638730 (PMC8021710; doi:10.3389/fgene.2021.638730)
Supplement: Supplementary file 1 [file Data_Sheet_1.pdf]

Supplementary materials for: "The history of gene hunting in hereditary spinocerebellar degeneration: lessons from the past and future perspectives" by Yahia & Stevanin.

Supplementary Table 1. Methods used in identifying the subtypes of hereditary spinocerebellar disorders, excluding syndromic disorders in which ataxia and/or spastic paraplegia are not prominent clinical features and unconfirmed clinico-genetic entities (SPG59, 60, 65, 67, 68, 69, 70 and 71).

SPG: spastic paraplegia; LEMSPAD: leukoencephalopathy, motor delay, spasticity, and dysarthria syndrome; SCA: spinocerebellar ataxia; EA: episodic ataxia; SCA42ND: spinocerebellar ataxia type 42, early-onset, severe, with neurodevelopmental deficits; SPAX: spastic ataxia; DRPLA: dentatorubral-pallidoluysian atrophy; ADCADN: cerebellar ataxia, deafness, and narcolepsy, autosomal dominant; CAPOS: cerebellar ataxia, areflexia, pes cavus, optic atrophy, and sensorineural hearing loss; BNHS: Boucher-Neuhauser syndrome; SPOAN: spastic paraplegia, optic atrophy, and neuropathy; NEDSWMA: neurodevelopmental disorder with progressive spasticity and brain white matter abnormalities; SPPRS: Spastic paraplegia and psychomotor retardation with or without seizures; HHH syndrome: Hyperornithinemia-hyperammonemia-homocitrullinemia syndrome; EAOH: ataxia, early-onset, with oculomotor apraxia and hypoalbuminemia; AT: ataxia-telangiectasia; FRDA: Friedreich ataxia; CANVAS: cerebellar ataxia, neuropathy, and vestibular areflexia syndrome; AOA: ataxia-oculomotor apraxia; MSS: Marinesco-Sjogren syndrome; VED: Ataxia with isolated vitamin E deficiency; PHARC: polyneuropathy, hearing loss, ataxia, retinitis pigmentosa, and cataract; ICRD: infantile cerebellar-retinal degeneration; ATCAY: ataxia, cerebellar, Cayman type; CAMRQ: cerebellar ataxia, mental retardation, and dysequilibrium syndrome; LKPAT: leukoencephalopathy with ataxia; SESAMES: seizures, sensorineural deafness, ataxia, mental retardation, and electrolyte imbalance syndrome; PTBHS: Poretti-Boltshauser syndrome; PACA: pancreatic and cerebellar agenesis; GDHS: Gordon Holmes syndrome; NADGP: neurodegeneration with ataxia, dystonia, and gaze palsy, childhood-onset; MTDPS7: Mitochondrial DNA depletion syndrome 7; SCAE: Spinocerebellar ataxia with epilepsy; SCAR: autosomal recessive spinocerebellar ataxia; SCAN: autosomal recessive spinocerebellar ataxia with axonal neuropathy; ISQMR: ichthyosis, spastic quadriplegia, and mental retardation; ASAT: sideroblastic anemia with ataxia; SCAX1: spinocerebellar ataxia, X-linked 1; FXTAS: fragile X tremor/ataxia syndrome; LA: linkage analysis; CA: candidate gene approach; WES: whole-exome sequencing; TE: targeted next generation sequencing; WGS: whole-genome sequencing; Repeat-exp: repeat expansion detection approaches. #: molecular cause known, %: molecular cause unknown.

**Disease**	**Gene**	**OMIM**	**Method**	**Reference**
Autosomal dominant/de novo				
SPG4	*SPAST*	# 182601	LA, CA	(Hazan et al. 1999)
SPG6	*NIPA1*	# 600363	LA, CA	(Rainier et al. 2003)
SPG8	*WASHC5*	# 603563	LA, CA	(Valdmanis et al. 2007)
SPG10	*KIF5A*	# 604187	LA, CA	(Reid et al. 2002)
SPG12	*RTN2*	# 604805	WES	(Montenegro et al. 2012)
SPG13	*HSPD1*	# 605280	LA, CA	(Hansen et al. 2002)
SPG17	*BSCL2*	# 270685	LA, CA	(Windpassinger et al. 2004)
SPG19		% 607152	LA	(Orlacchio et al. 2005)
SPG29		% 609727	LA	(Orlacchio et al. 2005)
SPG31	*REEP1*	# 610250	LA, CA	(Züchner et al. 2006)

SPG36		% 613096	LA	(Schüle et al. 2009)
SPG38		% 612335	LA	(Orlacchio et al. 2008)
SPG41		% 613364	LA	(Zhao et al. 2008)
SPG42	*SLC33A1*	# 612539	LA, CA	(Lin et al. 2008)
SPG73	*CPT1C*	# 616282	LA, WES	(Rinaldi et al. 2015)
SPG80	*UBAP1*	# 618418	WES	(Farazi Fard et al. 2019)
LEMSPAD	*EIF2AK1*	# 618878	WES	(Mao et al. 2020)
TUBB4A-SPG	*TUBB4A*		TE	(Sagnelli et al. 2016)
BICD2-SPG	*BICD2*		LA, WES, DNA array	(Oates et al. 2013)
SCA1	*ATXN1*	# 164400	LA, Repeat-exp	(Banfi et al. 1994)
SCA2	*ATXN2*	# 183090	LA, Repeat-exp	(Pulst et al. 1996)
SCA3	*ATXN3*	# 109150	LA, Repeat-exp	(Kawaguchi et al. 1994)
SCA4		% 600223	LA	(Hellenbroich et al. 2003)
SCA6	*CACNA1A*	# 183086	CA, Repeat-exp	(Zhuchenko et al. 1997)
EA2		# 108500	LA, CA	(Ophoff et al. 1996)
SCA7	*ATXN7*	# 164500	LA, Repeat-exp	(David et al. 1997)
SCA8	*ATXN8OS*	# 608768	Repeat-exp	(Koob et al. 1999)
	ATXN8		CA	(Moseley et al. 2006)
SCA9		% 612876		(Higgins et al. 1997)
SCA10	*ATXN10*	# 603516	LA, Repeat-exp	(Matsuura et al. 2000)
SCA11	*TTBK2*	# 604432	LA, CA	(Houlden et al. 2007)
SCA12	*PPP2R2B*	# 604326	LA, Repeat-exp	(Holmes et al. 1999)
SCA13	*KCNC3*	# 605259	LA, CA	(Waters et al. 2006)
SCA14	*PRKCG*	# 605361	LA, CA	(Chen et al. 2003)
SCA15	*ITPR1*	# 606658	LA, CA, DNA array	(Van De Leemput et al. 2007)
SCA29		# 117360	LA, WES	(Huang et al. 2012)
SCA17	*TBP*	# 607136	CA, Repeat-exp	(Reiji Koide et al. 1999)
SCA18		% 607458	LA	(Brkanac et al. 2002)
SCA19	*KCND3*	# 607346	LA, WES	(Lee et al. 2012)
SCA20		# 608687	LA, DNA array	(Knight et al. 2008)
SCA21	*TMEM240*	# 607454	LA, WES	(Delplanque et al. 2014)
SCA23	*PDYN*	# 610245	LA, CA	(Bakalkin et al. 2010)
SCA25		% 608703	LA	(Stevanin et al. 2004)
SCA26	*EEF2*	# 609306	LA, TE	(Hekman et al. 2012)
SCA27	*FGF14*	# 609307	LA, CA	(Van Swieten et al. 2003)
SCA30		% 613371	LA	(Storey et al. 2009)
SCA31	*BEAN1*	# 117210	LA, CA	(Sato et al. 2009)
SCA35	*TGM6*	# 613908	LA, WES, DNA array	(Wang et al. 2010)
SCA36	*NOP56*	# 614153	LA, CA	(Kobayashi et al. 2011)
SCA37	*DAB1*	# 615945	LA, TE, DNA array	(Seixas et al. 2017)
SCA38	*ELOVL5*	# 615957	LA, TE, DNA array	(Di Gregorio et al. 2014)
SCA40	*CCDC88C*	# 616053	WES	(Tsoi et al. 2014)
SCA41	*TRPC3*	# 616410	WES	(Fogel et al. 2015)

SCA42	*CACNA1G*	# 616795	LA, WES, DNA array	(Coutelier, Blesneac, et al. 2015)
SCA42ND		# 618087	WES	(Chemin et al. 2018)
SCA43	*MME*	# 617018	LA, WES, DNA array	(Depondt et al. 2016)
SCA45	*FAT2*	# 617769	WES	(Nibbeling et al. 2017)
SCA46	*PLD3*	# 617770	WES	(Nibbeling et al. 2017)
SCA47	*PUM1*	# 617931	CA, WES, DNA array	(Gennarino et al. 2018)
SPAX1	*VAMP1*	# 108600	LA, CA	(Bourassa et al. 2012)
KCNA2-SPG and ataxia	*KCNA2*		WES	(Helbig et al. 2016)
DRPLA	*ATN1*	# 125370	Repeat-exp	(R. Koide et al. 1994)
ADCADN	*DNMT1*	# 604121	WES	(Winkelmann et al. 2012)
CAPOS	*ATP1A3*	# 601338	WES	(Demos et al. 2014)
EA1	*KCNA1*	# 160120	LA, CA	(Browne et al. 1994)
EA3		% 606554	LA	(Steckley et al. 2001)
EA5	*CACNB4*	# 613855	CA	(Escayg et al. 2000)
EA6	*SLC1A3*	# 612656	CA	(Jen et al. 2005)
EA7		% 611907	LA	(Kerber et al. 2007)
EA8		% 616055	LA	(Conroy et al. 2014)
EA9	*SCN2A*	# 618924	CA	(Liao et al. 2010)
Autosomal Recessive				
SPG5A	*CYP7B1*	# 270800	LA, CA	(Tsaousidou et al. 2008)
SPG11	*SPG11*	# 604360	LA, CA	(Stevanin et al. 2007)
SPG14		% 605229	LA	(Vazza et al. 2000)
SPG15	*ZFYVE26*	# 270700	LA, CA	(Hanein et al. 2008)
SPG20	*SPG20*	# 275900	LA, CA	(Patel et al. 2002)
SPG21	*ACP33*	# 248900	LA, CA	(Simpson et al. 2003)
SPG23	*DSTYK*	# 270750	WES	(J. Y. W. Lee et al. 2017)
SPG24		% 607584	LA	(Hodgkinson et al. 2002)
SPG25		% 608220	LA	(Zortea et al. 2002)
SPG26	*B4GALNT1*	# 609195	LA, WES	(Boukhris et al. 2013)
SPG27		% 609041	LA	(Meijer et al. 2004)
SPG28	*DDHD1*	# 609340	LA, TE	(Tesson et al. 2012)
SPG32		% 611252	LA	(Stevanin et al. 2007)
SPG35	*FA2H*	# 612319	DNA array, CA	(Edvardson et al. 2008)
SPG39	*PNPLA6*	# 612020	LA, CA	(Rainier et al. 2008)
BNHS		# 215470	WES	(Synofzik et al. 2014)
SPG43	*C19orf12*	# 615043	WES	(Landouré et al. 2013)
SPG44	*GJC2*	# 613206	CA	(Orthmann-Murphy et al. 2009)
SPG45	*NT5C2*	# 613162	WES	(Novarino et al. 2014)
SPG46	*GBA2*	# 614409	WES	(Martin et al. 2013)
SPG47	*AP4B1*	# 614066	LA, CA	(Abou Jamra et al. 2011)
SPG48	*AP5Z1*	# 613647	CA	(Słabicki et al. 2010)

SPG49	*TECPR2*	# 615031	WES	(Oz-Levi et al. 2012)
SPG50	*AP4M1*	# 612936	LA, CA	(Verkerk et al. 2009)
SPG51	*AP4E1*	# 613744	DNA array	(Moreno-De-Luca et al. 2011)
SPG52	*AP4S1*	# 614067	LA, WES	(Abou Jamra et al. 2011)
SPG53	*VPS37A*	# 614898	LA, CA	(Zivony-Elboum et al. 2012)
SPG54	*DDHD2*	# 615033	WES	(Schuurs-Hoeijmakers et al. 2012)
SPG55	*C12orf65*	# 615035	LA, WES, DNA array	(Shimazaki et al. 2012)
SPG56	*CYP2U1*	# 615030	LA, CA, DNA array	(Tesson et al. 2012)
SPG57	*TFG*	# 615658	LA, WES, DNA array	(Beetz et al. 2013)
SPG61	*ARL6IP1*	# 615685	WES	(Novarino et al. 2014)
SPG62	*ERLIN1*	# 615681	WES	(Novarino et al. 2014)
SPG63	*AMPD2*	# 615686	WES	(Novarino et al. 2014)
SPG64	*ENTPD1*	# 615683	WES	(Novarino et al. 2014)
SPOAN	*KLC2*	# 609541	WGS	(Melo et al. 2015)
SPG74	*IBA57*	# 616451	LA, WES	(Lossos et al. 2015)
SPG75	*MAG*	# 616680	WES	(Novarino et al. 2014)
SPG76	*CAPN1*	# 616907	WES	(Gan-Or et al. 2016)
SPG77	*FARS2*	# 617046	WES, DNA array	(Yang et al. 2016)
SPG78	*ATP13A2*	# 617225	WES	(Kara et al. 2016)
SPG79	*UCHL1*	# 615491	WES, DNA array	(Bilguvar et al. 2013)
SPG81	*SELENOI*	# 618768	LA, DNA array, WES	(Ahmed et al. 2017)
SPG82	*PCYT2*	# 618770	WES	(Vaz et al. 2019)
SPG83	*HPDL*	# 619027	WES	(Husain et al. 2020)
NEDSWMA		# 619026	WES	(Husain et al. 2020)
Hereditary sensory neuropathy with spastic paraplegia	*CCT5*	# 256840	LA, CA	(Bouhouche et al. 2006)
CTX	*CYP27A1*	# 213700	CA	(Cali et al. 1991)
SPPRS	*HACE1*	# 616756	DNA array, WES	(Hollstein et al. 2015)
HHH syndrome	*SLC25A15*	# 238970	CA	(Camacho et al. 1999)
Argininemia	*ARG1*	# 207800	CA	(Haraguchi et al. 1990)
DARS2-SPG	*DARS2*		CA	(Lan et al. 2017)
PLA2G6-SPG	*PLA2G6*		WES, TE	(Ozes et al. 2017)
GPT2-SPG	*GPT2*		WES	(Kaymakcalan et al. 2018)
ADCY5-SPG	*ADCY5*		WES, TE	(Waalkens et al. 2018)
RNF170-SPG	*RNF170*		WES, WGS	(Wagner et al. 2019)
LYST-SPG	*LYST*		LA, WES, DNA array	(Shimazaki et al. 2014)

EAOH	*APTX*	# 208920	LA, CA	(Date et al. 2001)
AT	*ATM*	# 208900	LA, CA	(Savitsky et al. 1995)
AT-like disorder 1	*MRE11*	# 604391	CA	(Stewart et al. 1999)
Nijmegen breakage syndrome	*NBN*	# 251260	LA, CA	(Varon et al. 1998)
Cockayne syndrome, type A	*ERCC8*	# 216400	CA	(Henning et al. 1995)
Cockayne syndrome, type B	*ERCC6*	# 133540	CA	(Troelstra et al. 1992)
Leukoencephalopathy with vanishing white matter	*EIF2B1*	# 603896	CA	(Van Der Knaap et al. 2002)
	EIF2B2		LA, CA	(Leegwater et al. 2001)
	EIF2B3		CA	(Van Der Knaap et al. 2002)
	EIF2B4		CA	(Van Der Knaap et al. 2002)
	EIF2B5		LA, CA	(Leegwater et al. 2001)
FRDA	*FXN*	# 229300	LA, CA	(Campuzano et al. 1996)
HARS1-ataxia	*HARS1*		WES, TE	(Galatolo et al. 2020)
CANVAS	*RFC1*	# 614575	LA, DNA array, WGS	(Cortese et al. 2019)
SPAX2	*KIF1C*	# 611302	LA, DNA array, WES	(Dor et al. 2014)
SPAX3	*MARS2*	# 611390	LA, DNA array, CA	(Bayat et al. 2012)
SPAX6	*SACS*	# 270550	LA, CA	(Engert et al. 2000)
SPAX8	*NKX6-2*	# 617560	WES	(Chelban et al. 2017)
SPAX9	*CHP1*	# 618438	LA, DNA array, WES	(Mendoza-Ferreira et al. 2018)
POLR3A-SPAX	*POLR3A*		LA, DNA array, WES	(Minnerop et al. 2017)
AOA3	*PIK3R5*	# 615217	LA, DNA array, CA	(Tassan et al. 2012)
AOA4	*PNKP*	# 616267	DNA array, CA	(Bras et al. 2015)
MSS	*SIL1*	# 248800	LA, CA	(Anttonen et al. 2005)
VED	*TTPA*	# 277460	LA, CA	(Ouahchi et al. 1995)
PHARC	*ABHD12*	# 612674	LA, CA	(Fiskerstrand et al. 2010)
ICRD	*ACO2*	# 614559	LA, DNA array, WES	(Spiegel et al. 2012)
ATCAY	*ATCAY*	# 601238	LA, CA	(Bomar et al. 2003)
CAMRQ1	*VLDLR*	# 224050	LA, CA	(Boycott et al. 2005)
CAMRQ4	*ATP8A2*	# 615268	DNA array, TE	(Onat et al. 2013)
LKPAT	*CLCN2*	# 615651	WES	(Depienne et al. 2013)
SESAMES	*KCNJ10*	# 612780	LA, CA	(Scholl et al. 2009)
PTBHS	*LAMA1*	# 615960	DNA array, WES	(Aldinger et al. 2014)
PACA	*PTF1A*	# 609069	LA, CA	(Sellick et al. 2004)

GDHS	*RNF216*	# 212840	WES, TE	(Margolin et al. 2013)
NADGP	*SQSTM1*	# 617145	WES	(Haack et al. 2016)
PMM2-ataxia	*PMM2*		WES	(Van De Warrenburg et al. 2016)
SCAE	*POLG*	# 607459	CA	(Winterthun et al. 2005)
SCAR2	*PMPCA*	# 213200	LA, CA, WES	(Jobling et al. 2015)
SCAR3		% 271250	LA	(Bomont et al. 2000)
SCAR4	*VPS13D*	# 607317	LA, DNA array, WES	(Seong et al. 2018)
SCAR6		% 608029	LA	(Tranebjaerg et al. 2003)
SCAR7	*TPP1*	# 609270	LA, WES	(Sun et al. 2013)
SCAR8	*SYNE1*	# 610743	LA, CA	(Gros-Louis et al. 2007)
SCAR9	*ADCK3*	# 612016	CA	(Mollet et al. 2008)
SCAR10	*ANO10*	# 613728	LA, DNA array, TE	(Vermeer et al. 2010)
SCAR11	*SYT14*	# 614229	WES	(Doi et al. 2011)
SCAR12	*WWOX*	# 614322	WES	(Mallaret et al. 2014)
SCAR15	*RUBCN*	# 615705	LA, CA	(Assoum et al. 2010)
SCAR17	*CWF19L1*	# 616127	LA, DNA array, WES	(Burns et al. 2014)
SCAR19	*SLC9A1*	# 616291	DNA array, WES	(Guissart et al. 2015)
SCAR20	*SNX14*	# 616354	DNA array, WES	(Thomas et al. 2014)
SCAR21	*SCYL1*	# 616719	WES	(Schmidt et al. 2015)
SCAR22	*VWA3B*	# 616948	DNA array, WES	(Kawarai et al. 2016)
SCAR23	*TDP2*	# 616949	DNA array, WES	(Gómez-Herreros et al. 2014)
SCAR24	*UBA5*	# 617133	WES	(Duan et al. 2016)
SCAR25	*ATG5*	# 617584	LA, DNA array, WES	(Kim et al. 2016)
SCAR26	*XRCC1*	# 617633	WES	(Hoch et al. 2017)
SCAR27	*GDAP2*	# 618369	WES	(Eidhof et al. 2018)
SCAR28	*THG1L*	# 618800	WES	(Edvardson et al. 2016)
SCAN1	*TDP1*	# 607250	LA, CA	(Takashima et al. 2002)
SCAN2	*SETX*	# 606002	LA, CA	(Moreira et al. 2004)
SCAN3	*COA7*	# 618387	WES	(Lyons et al. 2016)
Dual autosomal dominant and recessive transmissions				
Dominant SPG3A	*ATL1*	# 182600	LA, CA	(Zhao et al. 2001)
ATL1-rcessive SPG			WES	(Khan et al. 2014)
Dominant SPG7	*PGN*	# 607259	CA	(Sánchez-Ferrero et al. 2013)
Recessive SPG7			LA, CA	(Casari et al. 1998)
SPG9A (dominant)	*ALDH18A1*	# 601162	LA, WES, TE, DNA array	(Coutelier et al. 2015)
SPG9B		# 616586		

(recessive)				
Recessive SPG18	*ERLIN2*	# 611225	LA, CA, DNA array	(Yildirim et al. 2011)
ERLIN2-dominant SPG			WES	(Rydning et al. 2018)
SPG30 (dominant)	*KIF1A*	# 610357	TE	(Ylikallio et al. 2015)
SPG30 (recessive)			WES, DNA array	(Erlich et al. 2011)
SPG72 (dominant)	*REEP2*	# 615625	LA, WES, DNA array	(Esteves et al. 2014)
REEP2-recessive SPG				
SCA5 (dominant)	*SPTBN2*	# 600224	LA, Repeat-exp	(Ikeda et al. 2006)
SCAR14 (recessive)		# 615386	TE	(Lise et al. 2012)
SCA28 (dominant)	*AFG3L2*	# 610246	LA, CA	(Di Bella et al. 2010)
SPAX5 (recessive)		# 614487	WES	(Pierson et al. 2011)
SCA34 (dominant)	*ELOVL4*	# 133190	LA, WES	(Cadieux-Dion et al. 2014)
ISQMR (recessive)		# 614457	WES, DNA array	(Aldahmesh et al. 2011)
SCA44 (dominant)	*GRM1*	# 617691	WES	(Watson et al. 2017)
SCAR13 (recessive)		# 614831	LA, WES	(Guergueltcheva et al. 2012)
SCA48 (dominant)	*STUB1*	# 618093	LA, WES	(Genis et al. 2018)
SCAR16 (recessive)		# 615768	LA, WES, DNA array	(Shi et al. 2013)
GRID2-ataxia (dominant)	*GRID2*		LA, TE, WES, DNA array	(Coutelier et al. 2015)
SCAR18 (recessive)		# 616204	DNA array	(Utine et al. 2013)
X-linked				
SPG1	*L1CAM*	# 303350	LA, CA	(Jouet et al. 1994)
SPG2	*PLP1*	# 312920	LA, CA	(Saugier-Veber et al. 1994)
SPG16		% 300266	LA	(Steinmuller et al. 1997)
SPG22	*SLC16A2*	# 300523	CA	(Friesema et al. 2004)
SPG34		% 300750	LA	(Macedo-Souza et al. 2008)
ASAT	*ABCB7*	# 301310	LA, CA	(Allikmets et al. 1999)
SCAX1	*ATP2B3*	# 302500	TE	(Zanni et al. 2012)
FXTAS	*FMR1*	# 300623	CA	(Hagerman et al. 2001)

Mitochondrial				
SPAX4	*MTPAP*	# 613672	LA, DNA array, CA	(Crosby et al. 2010)
Mitochondrial complex V deficiency	*MTATP6*		CA	(Verny et al. 2011)
MTDPS7	*TWNK*	# 271245	CA	(Sarzi et al. 2007)

References

Abou Jamra, Rami, Orianne Philippe, Annick Raas-Rothschild, Sebastian H. Eck, Elisabeth Graf, Rebecca Buchert, et al. 2011. Adaptor Protein Complex 4 Deficiency Causes Severe Autosomal-Recessive Intellectual Disability, Progressive Spastic Paraplegia, Shy Character, and Short Stature. *American Journal of Human Genetics* 88 (6): 788–95. https://doi.org/10.1016/j.ajhg.2011.04.019.

Ahmed, Mustafa Y., Aisha Al-Khayat, Fathiya Al-Murshedi, Amna Al-Futaisi, Barry A. Chioza, J. Pedro Fernandez-Murray, et al. 2017. A Mutation of EPT1 (SELENOI) Underlies a New Disorder of Kennedy Pathway Phospholipid Biosynthesis. *Brain* 140 (3): 547–54. https://doi.org/10.1093/brain/aww318.

Aldahmesh, Mohammed A., Jawahir Y. Mohamed, Hisham S. Alkuraya, Ishwar C. Verma, Ratna D. Puri, Ayodele A. Alaiya, et al. 2011. Recessive Mutations in ELOVL4 Cause Ichthyosis, Intellectual Disability, and Spastic Quadriplegia. *American Journal of Human Genetics* 89 (6): 745–50. https://doi.org/10.1016/j.ajhg.2011.10.011.

Aldinger, Kimberly A., Stephen J. Mosca, Martine Tétreault, Jennifer C. Dempsey, Gisele E. Ishak, Taila Hartley, et al. 2014. Mutations in LAMA1 Cause Cerebellar Dysplasia and Cysts with and without Retinal Dystrophy. *American Journal of Human Genetics* 95 (2): 227–34. https://doi.org/10.1016/j.ajhg.2014.07.007.

Allikmets, Rando, Wendy H. Raskind, Amy Hutchinson, Nichole D. Schueck, Michael Dean, and David M. Koeller. 1999. Mutation of a Putative Mitochondrial Iron Transporter Gene (ABC7) in X-Linked Sideroblastic Anemia and Ataxia (XLSA/A). *Human Molecular Genetics* 8 (5): 743–49. https://doi.org/10.1093/hmg/8.5.743.

Anttonen, Anna Kaisa, Ibrahim Mahjneh, Riikka H. Hämäläinen, Clotilde Lagier-Tourenne, Outi Kopra, Laura Waris, et al. 2005. The Gene Disrupted in Marinesco-Sjögren Syndrome Encodes SIL1, an HSPA5 Cochaperone. *Nature Genetics* 37 (12): 1309–11. https://doi.org/10.1038/ng1677.

Assoum, Mirna, Mustafa A. Salih, Nathalie Drouot, Brahim Dorra H'Mida-Ben, Clotilde Lagier-Tourenne, Abdulmajeed Aldrees, et al. 2010. Rundataxin, a Novel Protein with RUN and Diacylglycerol Binding Domains, Is Mutant in a New Recessive Ataxia. *Brain* 133 (8): 2439–47. https://doi.org/10.1093/brain/awq181.

Bakalkin, Georgy, Hiroyuki Watanabe, Justyna Jezierska, Cloë Depoorter, Corien Verschuuren-Bemelmans, Igor Bazov, et al. 2010. Prodynorphin Mutations Cause the Neurodegenerative Disorder Spinocerebellar Ataxia Type 23. *American Journal of Human Genetics* 87 (5):

593–603. https://doi.org/10.1016/j.ajhg.2010.10.001.

Banfi, Sandro, Antonio Servadio, Ming yi Chung, Thomas J. Kwiatkowski, Alanna E. McCall, Lisa A. Duvick, et al. 1994. Identification and Characterization of the Gene Causing Type 1 Spinocerebellar Ataxia. *Nature Genetics* 7 (4): 513–20. https://doi.org/10.1038/ng0894-513.

Bayat, Vafa, Isabelle Thiffault, Manish Jaiswal, Martine Tétreault, Taraka Donti, Florin Sasarman, et al. 2012. Mutations in the Mitochondrial Methionyl-TRNA Synthetase Cause a Neurodegenerative Phenotype in Flies and a Recessive Ataxia (ARSAL) in Humans. *PLoS Biology* 10 (3). https://doi.org/10.1371/journal.pbio.1001288.

Beetz, Christian, Adam Johnson, Amber L. Schuh, Seema Thakur, Rita Eva Varga, Thomas Fothergill, et al. 2013. Inhibition of TFG Function Causes Hereditary Axon Degeneration by Impairing Endoplasmic Reticulum Structure. *Proceedings of the National Academy of Sciences of the United States of America* 110 (13): 5091–96. https://doi.org/10.1073/pnas.1217197110.

Bella, Daniela Di, Federico Lazzaro, Alfredo Brusco, Massimo Plumari, Giorgio Battaglia, Annalisa Pastore, et al. 2010. Mutations in the Mitochondrial Protease Gene AFG3L2 Cause Dominant Hereditary Ataxia SCA28. *Nature Genetics* 42 (4): 313–21. https://doi.org/10.1038/ng.544.

Bilguvar, Kaya, Navneet K. Tyagi, Cigdem Ozkara, Beyhan Tuysuz, Mehmet Bakircioglu, Murim Choi, et al. 2013. Recessive Loss of Function of the Neuronal Ubiquitin Hydrolase UCHL1 Leads to Early-Onset Progressive Neurodegeneration. *Proceedings of the National Academy of Sciences of the United States of America* 110 (9): 3489–94. https://doi.org/10.1073/pnas.1222732110.

Bomar, Jamee M., Paul J. Benke, Eric L. Slattery, Radhika Puttagunta, Larry P. Taylor, Eunju Seong, et al. 2003. Mutations in a Novel Gene Encoding a CRAL-TRIO Domain Cause Human Cayman Ataxia and Ataxia/Dystonia in the Jittery Mouse. *Nature Genetics* 35 (3): 264–69. https://doi.org/10.1038/ng1255.

Bomont, Pascale, Mitsunory Watanabe, Ruth Gershoni-Barush, Masami Shizuka, Makoto Tanaka, Jinpei Sugano, et al. 2000. Homozygosity Mapping of Spinocerebellar Ataxia with Cerebellar Atrophy and Peripheral Neuropathy to 9q33-34, and with Hearing Impairment and Optic Atrophy to 6p21-23. *European Journal of Human Genetics* 8 (12): 986–90. https://doi.org/10.1038/sj.ejhg.5200586.

Bouhouche, A., A. Benomar, N. Bouslam, T. Chkili, and M. Yahyaoui. 2006. Mutation in the Epsilon Subunit of the Cytosolic Chaperonin-Containing t-Complex Peptide-1 (Cct5) Gene Causes Autosomal Recessive Mutilating Sensory Neuropathy with Spastic Paraplegia. *Journal of Medical Genetics* 43 (5): 441–43. https://doi.org/10.1136/jmg.2005.039230.

Boukhris, Amir, Rebecca Schule, José L. Loureiro, Charles Marques Lourenço, Emeline Mundwiller, Michael A. Gonzalez, et al. 2013. Alteration of Ganglioside Biosynthesis Responsible for Complex Hereditary Spastic Paraplegia. *American Journal of Human Genetics* 93 (1): 118–23. https://doi.org/10.1016/j.ajhg.2013.05.006.

Bourassa, Cynthia V., Inge A. Meijer, Nancy D. Merner, Kanwal K. Grewal, Mark G. Stefanelli, Kathleen Hodgkinson, et al. 2012. VAMP1 Mutation Causes Dominant Hereditary Spastic Ataxia in Newfoundland Families. *American Journal of Human Genetics* 91 (3): 548–52. https://doi.org/10.1016/j.ajhg.2012.07.018.

Boycott, Kym M., Shauna Flavelle, Alexandre Bureau, Hannah C. Glass, T. Mary Fujiwara, Elaine Wirrell, et al. 2005. Homozygous Deletion of the Very Low Density Lipoprotein Receptor Gene Causes Autosomal Recessive Cerebellar Hypoplasia with Cerebral Gyral Simplification. *American Journal of Human Genetics* 77 (3): 477–83. https://doi.org/10.1086/444400.

Bras, Jose, Isabel Alonso, Clara Barbot, Maria Manuela Costa, Lee Darwent, Tatiana Orme, et al. 2015. Mutations in PNKP Cause Recessive Ataxia with Oculomotor Apraxia Type 4. *American Journal of Human Genetics* 96 (3): 474–79. https://doi.org/10.1016/j.ajhg.2015.01.005.

Brkanac, Zoran, Magali Fernandez, Mark Matsushita, Hilary Lipe, John Wolff, Thomas D. Bird, et al. 2002. Autosomal Dominant Sensory/Motor Neuropathy with Ataxia (SMNA): Linkage to Chromosome 7q22-Q32. *American Journal of Medical Genetics - Neuropsychiatric Genetics* 114 (4): 450–57. https://doi.org/10.1002/ajmg.10361.

Browne, David L., Stephen T. Gancher, John G. Nutt, Ewout R.P. Brunt, Eric A. Smith, Patricia Kramer, et al. 1994. Episodic Ataxia/Myokymia Syndrome Is Associated with Point Mutations in the Human Potassium Channel Gene, KCNA1. *Nature Genetics* 8 (2): 136–40. https://doi.org/10.1038/ng1094-136.

Burns, Randi, Karen Majczenko, Jishu Xu, Weiping Peng, Zuhal Yapici, James J. Dowling, et al. 2014. Homozygous Splice Mutation in CWF19L1 in a Turkish Family with Recessive Ataxia Syndrome. *Neurology* 83 (23): 2175–82. https://doi.org/10.1212/WNL.0000000000001053.

Cadieux-Dion, Maxime, Maude Turcotte-Gauthier, Anne Noreau, Caroline Martin, Caroline Meloche, Micheline Gravel, et al. 2014. Expanding the Clinical Phenotype Associated with ELOVL4 Mutation: Study of a Large French-Canadian Family with Autosomal Dominant Spinocerebellar Ataxia and Erythrokeratodermia. *JAMA Neurology* 71 (4): 470–75. https://doi.org/10.1001/jamaneurol.2013.6337.

Cali, J. J., C. L. Hsieh, U. Francke, and D. W. Russell. 1991. Mutations in the Bile Acid Biosynthetic Enzyme Sterol 27-Hydroxylase Underlie Cerebrotendinous Xanthomatosis. *Journal of Biological Chemistry* 266 (12): 7779–83.

Camacho, Jose A., Cassandra Obie, Barbara Biery, Barbara K. Goodman, Chien An Hu, Shlomo Almashanu, et al. 1999. Hyperornithinaemia-Hyperammonaemia-Homocitrullinuria Syndrome Is Caused by Mutations in a Gene Encoding a Mitochondrial Ornithine Transporter. *Nature Genetics* 22 (2): 151–58. https://doi.org/10.1038/9658.

Campuzano, Victoria, Laura Montermini, Maria Dolores Moltò, Luigi Pianese, Mireille Cossée, Francesca Cavalcanti, et al. 1996. Friedreich's Ataxia: Autosomal Recessive Disease Caused by an Intronic GAA Triplet Repeat Expansion. *Science* 271 (5254): 1423–27.

https://doi.org/10.1126/science.271.5254.1423.

Casari, Giorgio, Maurizio De Fusco, Sonia Ciarmatori, Massimo Zeviani, Marina Mora, Patricio Fernandez, et al. 1998. Spastic Paraplegia and OXPHOS Impairment Caused by Mutations in Paraplegin, a Nuclear-Encoded Mitochondrial Metalloprotease. *Cell* 93 (6): 973–83. https://doi.org/10.1016/S0092-8674(00)81203-9.

Chelban, Viorica, Nisha Patel, Jana Vandrovcova, M. Natalia Zanetti, David S. Lynch, Mina Ryten, et al. 2017. Mutations in NKX6-2 Cause Progressive Spastic Ataxia and Hypomyelination. *American Journal of Human Genetics* 100 (6): 969–77. https://doi.org/10.1016/j.ajhg.2017.05.009.

Chemin, Jean, Karine Siquier-Pernet, Michaël Nicouleau, Giulia Barcia, Ali Ahmad, Daniel Medina-Cano, et al. 2018. De Novo Mutation Screening in Childhood-Onset Cerebellar Atrophy Identifies Gain-of-Function Mutations in the CACNA1G Calcium Channel Gene. *Brain* 141 (7): 1998–2013. https://doi.org/10.1093/brain/awy145.

Chen, Dong Hui, Zoran Brkanac, Christophe L.M.J. Verlinde, Xiao Jian Tan, Laura Bylenok, David Nochlin, et al. 2003. Missense Mutations in the Regulatory Domain of PKCγ: A New Mechanism for Dominant Nonepisodic Cerebellar Ataxia. *American Journal of Human Genetics* 72 (4): 839–49. https://doi.org/10.1086/373883.

Conroy, Judith, Paul McGettigan, Raymond Murphy, David Webb, Sinéad M. Murphy, Blathnaid McCoy, et al. 2014. A Novel Locus for Episodic Ataxia: UBR4 the Likely Candidate. *European Journal of Human Genetics* 22 (4): 505–10. https://doi.org/10.1038/ejhg.2013.173.

Cortese, Andrea, Roberto Simone, Roisin Sullivan, Jana Vandrovcova, Huma Tariq, Yau Way Yan, et al. 2019. Biallelic Expansion of an Intronic Repeat in RFC1 Is a Common Cause of Late-Onset Ataxia. *Nature Genetics* 51 (4): 649–58. https://doi.org/10.1038/s41588-019-0372-4.

Coutelier, Marie, Iulia Blesneac, Arnaud Monteil, Marie Lorraine Monin, Kunie Ando, Emeline Mundwiller, et al. 2015. A Recurrent Mutation in CACNA1G Alters Cav3.1 T-Type Calcium-Channel Conduction and Causes Autosomal-Dominant Cerebellar Ataxia. *American Journal of Human Genetics* 97 (5): 726–37. https://doi.org/10.1016/j.ajhg.2015.09.007.

Coutelier, Marie, Lydie Burglen, Emeline Mundwiller, Myriam Abada-Bendib, Diana Rodriguez, Sandra Chantot-Bastaraud, et al. 2015. GRID2 Mutations Span from Congenital to Mild Adult-Onset Cerebellar Ataxia. *Neurology* 84 (17): 1751–59. https://doi.org/10.1212/WNL.0000000000001524.

Coutelier, Marie, Cyril Goizet, Alexandra Durr, Florence Habarou, Sara Morais, Alexandre Dionne-Laporte, et al. 2015. Alteration of Ornithine Metabolism Leads to Dominant and Recessive Hereditary Spastic Paraplegia. *Brain* 138 (8): 2191–2205. https://doi.org/10.1093/brain/awv143.

Crosby, Andrew H., Heema Patel, Barry A. Chioza, Christos Proukakis, Kay Gurtz, Michael A.

Patton, et al. 2010. Defective Mitochondrial Mrna Maturation Is Associated with Spastic Ataxia. *American Journal of Human Genetics* 87 (5): 655–60. https://doi.org/10.1016/j.ajhg.2010.09.013.

Date, Hidetoshi, Osamu Onodera, Hajime Tanaka, Kiyoshi Iwabuchi, Kazutoshi Uekawa, Shuichi Igarashi, et al. 2001. Early-Onset Ataxia with Ocular Motor Apraxia and Hypoalbuminemia Is Caused by Mutations in a New HIT Superfamily Gene. *Nature Genetics* 29 (2): 184–88. https://doi.org/10.1038/ng1001-184.

David, Gilles, Nacer Abbas, Giovanni Stevanin, Alexandra Dürr, Gaël Yvert, Géraldine Cancel, et al. 1997. Cloning of the SCA7 Gene Reveals a Highly Unstable CAG Repeat Expansion. *Nature Genetics* 17 (1): 65–70. https://doi.org/10.1038/ng0997-65.

Delplanque, Jérôme, David Devos, Vincent Huin, Alexandre Genet, Olivier Sand, Caroline Moreau, et al. 2014. TMEM240 Mutations Cause Spinocerebellar Ataxia 21 with Mental Retardation and Severe Cognitive Impairment. *Brain* 137 (10): 2657–63. https://doi.org/10.1093/brain/awu202.

Demos, Michelle K., Clara D.M. Van Karnebeek, Colin J.D. Ross, Shelin Adam, Yaoqing Shen, Shing Hei Zhan, et al. 2014. A Novel Recurrent Mutation in ATP1A3 Causes CAPOS Syndrome. *Orphanet Journal of Rare Diseases* 9 (1). https://doi.org/10.1186/1750-1172-9-15.

Depienne, Christel, Marianna Bugiani, Céline Dupuits, Damien Galanaud, Valérie Touitou, Nienke Postma, et al. 2013. Brain White Matter Oedema Due to ClC-2 Chloride Channel Deficiency: An Observational Analytical Study. *The Lancet Neurology* 12 (7): 659–68. https://doi.org/10.1016/S1474-4422(13)70053-X.

Depondt, Chantal, Simona Donatello, Myriam Rai, François Charles Wang, Mario Manto, Nicolas Simonis, et al. 2016. MME Mutation in Dominant Spinocerebellar Ataxia with Neuropathy (SCA43). *Neurology: Genetics* 2 (5). https://doi.org/10.1212/NXG.0000000000000094.

Doi, Hiroshi, Kunihiro Yoshida, Takao Yasuda, Mitsunori Fukuda, Yoko Fukuda, Hiroshi Morita, et al. 2011. Exome Sequencing Reveals a Homozygous SYT14 Mutation in Adult-Onset, Autosomal-Recessive Spinocerebellar Ataxia with Psychomotor Retardation. *American Journal of Human Genetics* 89 (2): 320–27. https://doi.org/10.1016/j.ajhg.2011.07.012.

Dor, Talya, Yuval Cinnamon, Laure Raymond, Avraham Shaag, Naima Bouslam, Ahmed Bouhouche, et al. 2014. KIF1C Mutations in Two Families with Hereditary Spastic Paraparesis and Cerebellar Dysfunction. *Journal of Medical Genetics* 51 (2): 137–42. https://doi.org/10.1136/jmedgenet-2013-102012.

Duan, Ranhui, Yuting Shi, Li Yu, Gehan Zhang, Jia Li, Yunting Lin, et al. 2016. UBA5 Mutations Cause a New Form of Autosomal Recessive Cerebellar Ataxia. *PLoS ONE* 11 (2). https://doi.org/10.1371/journal.pone.0149039.

Edvardson, Simon, Yael Elbaz-Alon, Chaim Jalas, Ashanti Matlock, Krishna Patel, Katherine

Labbé, et al. 2016. A Mutation in the THG1L Gene in a Family with Cerebellar Ataxia and Developmental Delay. *Neurogenetics* 17 (4): 219–25. https://doi.org/10.1007/s10048-016-0487-z.

Edvardson, Simon, Hiroko Hama, Avraham Shaag, John Moshe Gomori, Itai Berger, Dov Soffer, et al. 2008. Mutations in the Fatty Acid 2-Hydroxylase Gene Are Associated with Leukodystrophy with Spastic Paraparesis and Dystonia. *American Journal of Human Genetics* 83 (5): 643–48. https://doi.org/10.1016/j.ajhg.2008.10.010.

Eidhof, Ilse, Jonathan Baets, Erik Jan Kamsteeg, Tine Deconinck, Lisa Van Ninhuijs, Jean Jacques Martin, et al. 2018. GDAP2 Mutations Implicate Susceptibility to Cellular Stress in a New Form of Cerebellar Ataxia. *Brain* 141 (9): 2592–2604. https://doi.org/10.1093/brain/awy198.

Engert, James C., Pierre Bérubé, Jocelyne Mercier, Carole Doré, Pierre Lepage, Bing Ge, et al. 2000. ARSACS, a Spastic Ataxia Common in Northeastern Quebec, Is Caused by Mutations in a New Gene Encoding an 11.5-Kb ORF. *Nature Genetics* 24 (2): 120–25. https://doi.org/10.1038/72769.

Erlich, Yaniv, Simon Edvardson, Emily Hodges, Shamir Zenvirt, Pramod Thekkat, Avraham Shaag, et al. 2011. Exome Sequencing and Disease-Network Analysis of a Single Family Implicate a Mutation in KIF1A in Hereditary Spastic Paraparesis. *Genome Research* 21 (5): 658–64. https://doi.org/10.1101/gr.117143.110.

Escayg, Andrew, Michel De Waard, David D. Lee, Delphine Bichet, Peter Wolf, Thomas Mayer, et al. 2000. Coding and Noncoding Variation of the Human Calcium-Channel B4-Subunit Gene CACNB4 Patients with Idiopathic Generalized Epilepsy and Episodic Ataxia. *American Journal of Human Genetics* 66 (5): 1531–39. https://doi.org/10.1086/302909.

Esteves, Typhaine, Alexandra Durr, Emeline Mundwiller, José L. Loureiro, Maxime Boutry, Michael A. Gonzalez, et al. 2014. Loss of Association of REEP2 with Membranes Leads to Hereditary Spastic Paraplegia. *American Journal of Human Genetics* 94 (2): 268–77. https://doi.org/10.1016/j.ajhg.2013.12.005.

Farazi Fard, Mohammad Ali, Adriana P. Rebelo, Elena Buglo, Hamid Nemati, Hassan Dastsooz, Ina Gehweiler, et al. 2019. Truncating Mutations in UBAP1 Cause Hereditary Spastic Paraplegia. *American Journal of Human Genetics* 104 (4): 767–73. https://doi.org/10.1016/j.ajhg.2019.03.001.

Fiskerstrand, Torunn, Dorra H'Mida-Ben Brahim, Stefan Johansson, Abderrahim M'Zahem, Bjørn Ivar Haukanes, Nathalie Drouot, et al. 2010. Mutations in ABHD12 Cause the Neurodegenerative Disease PHARC: An Inborn Error of Endocannabinoid Metabolism. *American Journal of Human Genetics* 87 (3): 410–17. https://doi.org/10.1016/j.ajhg.2010.08.002.

Fogel, Brent L., Sonya M. Hanson, and Esther B.E. Becker. 2015. Do Mutations in the Murine Ataxia Gene TRPC3 Cause Cerebellar Ataxia in Humans? *Movement Disorders*. John Wiley and Sons Inc. https://doi.org/10.1002/mds.26096.

Friesema, Edith C.H., Prof Annette Grueters, Heike Biebermann, Heiko Krude, Arpad Von Moers, Maarten Reeser, et al. 2004. Association between Mutations in a Thyroid Hormone Transporter and Severe X-Linked Psychomotor Retardation. *Lancet* 364 (9443): 1435–37. https://doi.org/10.1016/S0140-6736(04)17226-7.

Galatolo, Daniele, Molly E. Kuo, Patrick Mullen, Rebecca Meyer-Schuman, Stefano Doccini, Roberta Battini, et al. 2020. Bi-Allelic Mutations in HARS1 Severely Impair Histidyl-TRNA Synthetase Expression and Enzymatic Activity Causing a Novel Multisystem Ataxic Syndrome. *Human Mutation* 41 (7): 1232–37. https://doi.org/10.1002/humu.24024.

Gan-Or, Ziv, Naima Bouslam, Nazha Birouk, Alexandra Lissouba, Daniel B. Chambers, Julie Vérièpe, et al. 2016. Mutations in CAPN1 Cause Autosomal-Recessive Hereditary Spastic Paraplegia. *American Journal of Human Genetics* 98 (5): 1038–46. https://doi.org/10.1016/j.ajhg.2016.04.002.

Genis, David, Sara Ortega-Cubero, Hector San Nicolas, Jordi Corral, Josep Gardenyes, Laura De Jorge, et al. 2018. Heterozygous STUB1 Mutation Causes Familial Ataxia with Cognitive Affective Syndrome (SCA48). *Neurology* 91 (21): E1988–98. https://doi.org/10.1212/WNL.0000000000006550.

Gennarino, Vincenzo A., Elizabeth E. Palmer, Laura M. McDonell, Li Wang, Carolyn J. Adamski, Amanda Koire, et al. 2018. A Mild PUM1 Mutation Is Associated with Adult-Onset Ataxia, Whereas Haploinsufficiency Causes Developmental Delay and Seizures. *Cell* 172 (5): 924-936.e11. https://doi.org/10.1016/j.cell.2018.02.006.

Gómez-Herreros, Fernando, Janneke H.M. Schuurs-Hoeijmakers, Mark McCormack, Marie T. Greally, Stuart Rulten, Rocío Romero-Granados, et al. 2014. TDP2 Protects Transcription from Abortive Topoisomerase Activity and Is Required for Normal Neural Function. *Nature Genetics* 46 (5): 516–21. https://doi.org/10.1038/ng.2929.

Gregorio, Eleonora Di, Barbara Borroni, Elisa Giorgio, Daniela Lacerenza, Marta Ferrero, Nicola Lo Buono, et al. 2014. ELOVL5 Mutations Cause Spinocerebellar Ataxia 38. *American Journal of Human Genetics* 95 (2): 209–17. https://doi.org/10.1016/j.ajhg.2014.07.001.

Gros-Louis, François, Nicolas Dupré, Patrick Dion, Michael A. Fox, Sandra Laurent, Steve Verreault, et al. 2007. Mutations in SYNE1 Lead to a Newly Discovered Form of Autosomal Recessive Cerebellar Ataxia. *Nature Genetics* 39 (1): 80–85. https://doi.org/10.1038/ng1927.

Guergueltcheva, Velina, Dimitar N. Azmanov, Dora Angelicheva, Katherine R. Smith, Teodora Chamova, Laura Florez, et al. 2012. Autosomal-Recessive Congenital Cerebellar Ataxia Is Caused by Mutations in Metabotropic Glutamate Receptor 1. *American Journal of Human Genetics* 91 (3): 553–64. https://doi.org/10.1016/j.ajhg.2012.07.019.

Guissart, Claire, Xiuju Li, Bruno Leheup, Nathalie Drouot, Bettina Montaut-Verient, Emmanuel Raffo, et al. 2015. Mutation of SLC9A1, Encoding the Major Na+/H+ Exchanger, Causes Ataxia-Deafness Lichtenstein-Knorr Syndrome. *Human Molecular Genetics* 24 (2): 463–70. https://doi.org/10.1093/hmg/ddu461.

Haack, Tobias B B., Arcangela Iuso, Laura S S. Kremer, Monika Hartig, Tim M M. Strom, Thomas Meitinger, et al. 2016. Absence of the Autophagy Adaptor SQSTM1/P62 Causes Childhood-Onset Neurodegeneration with Ataxia, Dystonia, and Gaze Palsy. *American Journal of Human Genetics* 99 (3): 735–43. https://doi.org/10.1016/j.ajhg.2016.06.026.

Hagerman, Randi J., M. Leehey, W. Heinrichs, F. Tassone, R. Wilson, J. Hills, et al. 2001. Intention Tremor, Parkinsonism, and Generalized Brain Atrophy in Male Carriers of Fragile X. *Neurology* 57 (1): 127–30. https://doi.org/10.1212/WNL.57.1.127.

Hanein, Sylvain, Elodie Martin, Amir Boukhris, Paula Byrne, Cyril Goizet, Abdelmadjid Hamri, et al. 2008. Identification of the SPG15 Gene, Encoding Spastizin, as a Frequent Cause of Complicated Autosomal-Recessive Spastic Paraplegia, Including Kjellin Syndrome. *American Journal of Human Genetics* 82 (4): 992–1002. https://doi.org/10.1016/j.ajhg.2008.03.004.

Hansen, Jens Jacob, Alexandra Dürr, Isabelle Cournu-Rebeix, Costa Georgopoulos, Debbie Ang, Marit Nyholm Nielsen, et al. 2002. Hereditary Spastic Paraplegia SPG13 Is Associated with a Mutation in the Gene Encoding the Mitochondrial Chaperonin Hsp60. *American Journal of Human Genetics* 70 (5): 1328–32. https://doi.org/10.1086/339935.

Haraguchi, Y., J. M.R. Aparicio, M. Takiguchi, I. Akaboshi, M. Yoshino, M. Mori, et al. 1990. Molecular Basis of Argininemia. Identification of Two Discrete Frame-Shift Deletions in the Liver-Type Arginase Gene. *Journal of Clinical Investigation* 86 (1): 347–50. https://doi.org/10.1172/JCI114707.

Hazan, Jamilé, Nùria Fonknechten, Delphine Mavel, Caroline Paternotte, Delphine Samson, François Artiguenave, et al. 1999. Spastin, a New AAA Protein, Is Altered in the Most Frequent Form of Autosomal Dominant Spastic Paraplegia. *Nature Genetics* 23 (3): 296–303. https://doi.org/10.1038/15472.

Hekman, Katherine E., Guo Yun Yu, Christopher D. Brown, Haipeng Zhu, Xiaofei Du, Kristina Gervin, et al. 2012. A Conserved EEF2 Coding Variant in SCA26 Leads to Loss of Translational Fidelity and Increased Susceptibility to Proteostatic Insult. *Human Molecular Genetics* 21 (26): 5472–83. https://doi.org/10.1093/hmg/dds392.

Helbig, Katherine L., Ulrike B.S. Hedrich, Deepali N. Shinde, Ilona Krey, Anne Christin Teichmann, Julia Hentschel, et al. 2016. A Recurrent Mutation in KCNA2 as a Novel Cause of Hereditary Spastic Paraplegia and Ataxia. *Annals of Neurology* 80 (4). https://doi.org/10.1002/ana.24762.

Hellenbroich, Yorck, S. Bubel, H. Pawlack, S. Opitz, P. Vieregge, E. Schwinger, et al. 2003. Refinement of the Spinocerebellar Ataxia Type 4 Locus in a Large German Family and Exclusion of CAG Repeat Expansions in This Region. *Journal of Neurology* 250 (6): 668–71. https://doi.org/10.1007/s00415-003-1052-x.

Henning, Karla A., Lei Li, Narayan Iyer, Lisa D. McDaniel, Michael S. Reagan, Randy Legerski, et al. 1995. The Cockayne Syndrome Group A Gene Encodes a WD Repeat Protein That Interacts with CSB Protein and a Subunit of RNA Polymerase II TFIIH. *Cell* 82 (4): 555–64. https://doi.org/10.1016/0092-8674(95)90028-4.

Higgins, J. J., L. T. Pho, S. E. Ide, L. E. Nee, and M. H. Polymeropoulos. 1997. Evidence for a New Spinocerebellar Ataxia Locus. *Movement Disorders* 12 (3): 412–17. https://doi.org/10.1002/mds.870120322.

Hoch, Nicolas C., Hana Hanzlikova, Stuart L. Rulten, Martine Tétreault, Emilia Komulainen, Limei Ju, et al. 2017. XRCC1 Mutation Is Associated with PARP1 Hyperactivation and Cerebellar Ataxia. *Nature* 541 (7635): 87–91. https://doi.org/10.1038/nature20790.

Hodgkinson, C. A., S. Bohlega, S. N. Abu-Amero, E. Cupler, M. Kambouris, B. F. Meyer, et al. 2002. A Novel Form of Autosomal Recessive Pure Hereditary Spastic Paraplegia Maps to Chromosome 13q14. *Neurology* 59 (12): 1905–9. https://doi.org/10.1212/01.WNL.0000036909.49629.21.

Hollstein, Ronja, David A. Parry, Lisa Nalbach, Clare V. Logan, Tim M. Strom, Verity L. Hartill, et al. 2015. HACE1 Deficiency Causes an Autosomal Recessive Neurodevelopmental Syndrome. *Journal of Medical Genetics* 52 (12): 797–803. https://doi.org/10.1136/jmedgenet-2015-103344.

Holmes, Susan E., Elizabeth E. O'Hearn, Melvin G. McInnis, Daniel A. Gorelick-Feldman, John J. Kleiderlein, Colleen Callahan, et al. 1999. Expansion of a Novel CAG Trinucleotide Repeat in the 5' Region of PPP2R2B Is Associated with SCA12. *Nature Genetics* 23 (4): 391–92. https://doi.org/10.1038/70493.

Houlden, Henry, Janel Johnson, Christopher Gardner-Thorpe, Tammaryn Lashley, Dena Hernandez, Paul Worth, et al. 2007. Mutations in TTBK2, Encoding a Kinase Implicated in Tau Phosphorylation, Segregate with Spinocerebellar Ataxia Type 11. *Nature Genetics* 39 (12): 1434–36. https://doi.org/10.1038/ng.2007.43.

Huang, Lijia, Jodi Warman Chardon, Melissa T. Carter, Kathie L. Friend, Tracy E. Dudding, Jeremy Schwartzentruber, et al. 2012. Missense Mutations in ITPR1 Cause Autosomal Dominant Congenital Nonprogressive Spinocerebellar Ataxia. *Orphanet Journal of Rare Diseases* 7 (1). https://doi.org/10.1186/1750-1172-7-67.

Husain, Ralf A., Mona Grimmel, Matias Wagner, J. Christopher Hennings, Christian Marx, René G. Feichtinger, et al. 2020. Bi-Allelic HPDL Variants Cause a Neurodegenerative Disease Ranging from Neonatal Encephalopathy to Adolescent-Onset Spastic Paraplegia. *American Journal of Human Genetics* 107 (2): 364–73. https://doi.org/10.1016/j.ajhg.2020.06.015.

Ikeda, Yoshio, Katherine A. Dick, Marcy R. Weatherspoon, Dan Gincel, Karen R. Armbrust, Joline C. Dalton, et al. 2006. Spectrin Mutations Cause Spinocerebellar Ataxia Type 5. *Nature Genetics* 38 (2): 184–90. https://doi.org/10.1038/ng1728.

Jen, J. C., J. Wan, T. P. Palos, B. D. Howard, and Robert W. Baloh. 2005. Mutation in the Glutamate Transporter EAAT1 Causes Episodic Ataxia, Hemiplegia, and Seizures. *Neurology* 65 (4): 529–34. https://doi.org/10.1212/01.WNL.0000172638.58172.5a.

Jobling, Rebekah K., Mirna Assoum, Oleksandr Gakh, Susan Blaser, Julian A. Raiman, Cyril Mignot, et al. 2015. PMPCA Mutations Cause Abnormal Mitochondrial Protein Processing in Patients with Non-Progressive Cerebellar Ataxia. *Brain* 138 (6): 1505–17.

https://doi.org/10.1093/brain/awv057.

Jouet, Monique, André Rosenthal, Giles Armstrong, John Macfarlane, Roger Stevenson, Joan Paterson, et al. 1994. X–Linked Spastic Paraplegia (SPG1), MASA Syndrome and X–Linked Hydrocephalus Result from Mutations in the L1 Gene. *Nature Genetics* 7 (3): 402–7. https://doi.org/10.1038/ng0794-402.

Kara, Eleanna, Arianna Tucci, Claudia Manzoni, David S. Lynch, Marilena Elpidorou, Conceicao Bettencourt, et al. 2016. Genetic and Phenotypic Characterization of Complex Hereditary Spastic Paraplegia. *Brain* 139 (7): 1904–18. https://doi.org/10.1093/brain/aww111.

Kawaguchi, Yoshiya, Toshihiro Okamoto, Masafumi Taniwaki, Megumi Aizawa, Miho Inoue, Sadao Katayama, et al. 1994. CAG Expansions in a Novel Gene for Machado-Joseph Disease at Chromosome 14q32.1. *Nature Genetics* 8 (3): 221–28. https://doi.org/10.1038/ng1194-221.

Kawarai, Toshitaka, Atsushi Tajima, Yukiko Kuroda, Naoki Saji, Antonio Orlacchio, Hideo Terasawa, et al. 2016. A Homozygous Mutation of VWA3B Causes Cerebellar Ataxia with Intellectual Disability. *Journal of Neurology, Neurosurgery and Psychiatry* 87 (6): 656–62. https://doi.org/10.1136/jnnp-2014-309828.

Kaymakcalan, Hande, Yanki Yarman, Nukte Goc, Fatih Toy, Cihan Meral, A. Gulhan Ercan-Sencicek, et al. 2018. Novel Compound Heterozygous Mutations in GPT2 Linked to Microcephaly, and Intellectual Developmental Disability with or without Spastic Paraplegia. *American Journal of Medical Genetics, Part A* 176 (2): 421–25. https://doi.org/10.1002/ajmg.a.38558.

Kerber, Kevin A., Joanna C. Jen, Hane Lee, Stanley F. Nelson, and Robert W. Baloh. 2007. A New Episodic Ataxia Syndrome with Linkage to Chromosome 19q13. *Archives of Neurology* 64 (5): 749–52. https://doi.org/10.1001/archneur.64.5.749.

Khan, Tahir Naeem, Joakim Klar, Muhammad Tariq, Shehla Anjum Baig, Naveed Altaf Malik, Raja Yousaf, et al. 2014. Evidence for Autosomal Recessive Inheritance in SPG3A Caused by Homozygosity for a Novel ATL1 Missense Mutation. *European Journal of Human Genetics* 22 (10): 1180–84. https://doi.org/10.1038/ejhg.2014.5.

Kim, Myungjin, Erin Sandford, Damian Gatica, Yu Qiu, Xu Liu, Yumei Zheng, et al. 2016. Mutation in ATG5 Reduces Autophagy and Leads to Ataxia with Developmental Delay. *ELife* 5 (January). https://doi.org/10.7554/elife.12245.

Knaap, Marjo S. Van Der, Peter A.J. Leegwater, Andrea A.M. Könst, Allerdien Visser, Sakkubai Naidu, Cees B.M. Oudejans, et al. 2002. Mutations in Each of the Five Subunits of Translation Initiation Factor EIF2B Can Cause Leukoencephalopathy with Vanishing White Matter. *Annals of Neurology* 51 (2): 264–70. https://doi.org/10.1002/ana.10112.

Knight, Melanie A., Dena Hernandez, Scott J. Diede, Hans G. Dauwerse, Ian Rafferty, Joyce Van de leemput, et al. 2008. A Duplication at Chromosome 11q12.2-11q12.3 Is Associated with Spinocerebellar Ataxia Type 20. *Human Molecular Genetics* 17 (24): 3847–53.

https://doi.org/10.1093/hmg/ddn283.

Kobayashi, Hatasu, Koji Abe, Tohru Matsuura, Yoshio Ikeda, Toshiaki Hitomi, Yuji Akechi, et al. 2011. Expansion of Intronic GGCCTG Hexanucleotide Repeat in NOP56 Causes SCA36, a Type of Spinocerebellar Ataxia Accompanied by Motor Neuron Involvement. *American Journal of Human Genetics* 89 (1): 121–30. https://doi.org/10.1016/j.ajhg.2011.05.015.

Koide, R., T. Ikeuchi, O. Onodera, H. Tanaka, S. Igarashi, K. Endo, et al. 1994. Unstable Expansion of CAG Repeat in Hereditary Dentatorubral–Pallidoluysian Atrophy (DRPLA). *Nature Genetics* 6 (1): 9–13. https://doi.org/10.1038/ng0194-9.

Koide, Reiji, Shigeichi Kobayashi, Takayoshi Shimohata, Takeshi Ikeuchi, Mieko Maruyama, Masaaki Saito, et al. 1999. A Neurological Disease Caused by an Expanded CAG Trinucleotide Repeat in the TATA-Binding Protein Gene: A New Polyglutamine Disease? *Human Molecular Genetics* 8 (11): 2047–53. https://doi.org/10.1093/hmg/8.11.2047.

Koob, Michael D., Melinda L. Moseley, Lawrence J. Schut, Kellie A. Benzow, Thomas D. Bird, John W. Day, et al. 1999. An Untranslated CTG Expansion Causes a Novel Form of Spinocerebellar Ataxia (SCA8). *Nature Genetics* 21 (4): 379–84. https://doi.org/10.1038/7710.

Lan, Min Yu, Yung Yee Chang, Tu Hseuh Yeh, Tsu Kung Lin, and Chin Song Lu. 2017. Leukoencephalopathy with Brainstem and Spinal Cord Involvement and Lactate Elevation (LBSL) with a Novel DARS2 Mutation and Isolated Progressive Spastic Paraparesis. *Journal of the Neurological Sciences*. Elsevier B.V. https://doi.org/10.1016/j.jns.2016.11.058.

Landouré, Guida, Peng Peng Zhu, Charles M. Lourenço, Janel O. Johnson, Camilo Toro, Katherine V. Bricceno, et al. 2013. Hereditary Spastic Paraplegia Type 43 (SPG43) Is Caused by Mutation in C19orf12. *Human Mutation* 34 (10): 1357–60. https://doi.org/10.1002/humu.22378.

Lee, John Y.W., Chao Kai Hsu, Magdalene Michael, Arti Nanda, Lu Liu, James R. McMillan, et al. 2017. Large Intragenic Deletion in DSTYK Underlies Autosomal-Recessive Complicated Spastic Paraparesis, SPG23. *American Journal of Human Genetics* 100 (2): 364–70. https://doi.org/10.1016/j.ajhg.2017.01.014.

Lee, Yi Chung, Alexandra Durr, Karen Majczenko, Yen Hua Huang, Yu Chao Liu, Cheng Chang Lien, et al. 2012. Mutations in KCND3 Cause Spinocerebellar Ataxia Type 22. *Annals of Neurology* 72 (6): 859–69. https://doi.org/10.1002/ana.23701.

Leegwater, Peter A.J., Gerre Vermeulen, Andrea A.M. Könst, Sakkubai Naidu, Joyce Mulders, Allerdien Visser, et al. 2001. Subunits of the Translation Initiation Factor ElF2B Are Mutant in Leukoencephalopathy with Vanishing White Matter. *Nature Genetics* 29 (4): 383–88. https://doi.org/10.1038/ng764.

Leemput, Joyce Van De, Jayanth Chandran, Melanie A. Knight, Lynne A. Holtzclaw, Sonja Scholz, Mark R. Cookson, et al. 2007. Deletion at ITPR1 Underlies Ataxia in Mice and Spinocerebellar Ataxia 15 in Humans. *PLoS Genetics* 3 (6): 1076–82.

https://doi.org/10.1371/journal.pgen.0030108.

Liao, Y., A. K. Anttonen, E. Liukkonen, E. Gaily, S. Maljevic, S. Schubert, et al. 2010. SCN2A Mutation Associated with Neonatal Epilepsy, Late-Onset Episodic Ataxia, Myoclonus, and Pain. *Neurology* 75 (16): 1454–58. https://doi.org/10.1212/WNL.0b013e3181f8812e.

Lin, Pengfei, Jianwei Li, Qiji Liu, Fei Mao, Jisheng Li, Rongfang Qiu, et al. 2008. A Missense Mutation in SLC33A1, Which Encodes the Acetyl-CoA Transporter, Causes Autosomal-Dominant Spastic Paraplegia (SPG42). *American Journal of Human Genetics* 83 (6): 752–59. https://doi.org/10.1016/j.ajhg.2008.11.003.

Lise, Stefano, Yvonne Clarkson, Emma Perkins, Alexandra Kwasniewska, Elham Sadighi Akha, Ricardo Parolin Schnekenberg, et al. 2012. Recessive Mutations in SPTBN2 Implicate β-III Spectrin in Both Cognitive and Motor Development. *PLoS Genetics* 8 (12). https://doi.org/10.1371/journal.pgen.1003074.

Lossos, Alexander, Claudia Stümpfig, Giovanni Stevanin, Marion Gaussen, Bat El Zimmerman, Emeline Mundwiller, et al. 2015. Fe/S Protein Assembly Gene IBA57 Mutation Causes Hereditary Spastic Paraplegia. *Neurology* 84 (7): 659–67. https://doi.org/10.1212/WNL.0000000000001270.

Lyons, Anabel Martinez, Anna Ardissone, Aurelio Reyes, Alan J. Robinson, Isabella Moroni, Daniele Ghezzi, et al. 2016. COA7 (C1orf163/RESA1) Mutations Associated with Mitochondrial Leukoencephalopathy and Cytochrome c Oxidase Deficiency. *Journal of Medical Genetics* 53 (12): 846–49. https://doi.org/10.1136/jmedgenet-2016-104194.

Macedo-Souza, Lúcia Inês, Fernando Kok, Silvana Santos, Luciana Licinio, Karina Lezirovitz, Rafaella M.P. Nascimento, et al. 2008. Reevaluation of a Large Family Defines a New Locus for X-Linked Recessive Pure Spastic Paraplegia (SPG34) on Chromosome Xq25. *Neurogenetics*. Neurogenetics. https://doi.org/10.1007/s10048-008-0130-8.

Mallaret, Martial, Matthis Synofzik, Jaeho Lee, Cari A. Sagum, Muhammad Mahajnah, Rajech Sharkia, et al. 2014. The Tumour Suppressor Gene WWOX Is Mutated in Autosomal Recessive Cerebellar Ataxia with Epilepsy and Mental Retardation. *Brain* 137 (2): 411–19. https://doi.org/10.1093/brain/awt338.

Mao, Dongxue, Chloe M. Reuter, Maura R.Z. Ruzhnikov, Anita E. Beck, Emily G. Farrow, Lisa T. Emrick, et al. 2020. De Novo EIF2AK1 and EIF2AK2 Variants Are Associated with Developmental Delay, Leukoencephalopathy, and Neurologic Decompensation. *American Journal of Human Genetics* 106 (4): 570–83. https://doi.org/10.1016/j.ajhg.2020.02.016.

Margolin, David H., Maria Kousi, Yee Ming Chan, Elaine T. Lim, Jeremy D. Schmahmann, Marios Hadjivassiliou, et al. 2013. Ataxia, Dementia, and Hypogonadotropism Caused by Disordered Ubiquitination. *New England Journal of Medicine* 368 (21): 1992–2003. https://doi.org/10.1056/NEJMoa1215993.

Martin, Elodie, Rebecca Schüle, Katrien Smets, Agnès Rastetter, Amir Boukhris, José L. Loureiro, et al. 2013. Loss of Function of Glucocerebrosidase GBA2 Is Responsible for Motor Neuron Defects in Hereditary Spastic Paraplegia. *American Journal of Human*

Genetics 92 (2): 238–44. https://doi.org/10.1016/j.ajhg.2012.11.021.

Matsuura, Tohru, Takanori Yamagata, Daniel L. Burgess, Astrid Rasmussen, Raji P. Grewal, Kei Watase, et al. 2000. Large Expansion of the ATTCT Pentanucleotide Repeat in Spinocerebellar Ataxia Type 10. *Nature Genetics* 26 (2): 191–94. https://doi.org/10.1038/79911.

Meijer, Inge A., Patrick Cossette, Julie Roussel, Melanie Benard, Sylvie Toupin, and Guy A. Rouleau. 2004. A Novel Locus for Pure Recessive Hereditary Spastic Paraplegia Maps to 10q22.1-10q24.1. *Annals of Neurology* 56 (4): 579–82. https://doi.org/10.1002/ana.20239.

Melo, Uirá S., Lucia I. Macedo-Souza, Thalita Figueiredo, Alysson R. Muotri, Joseph G. Gleeson, Gabriela Coux, et al. 2015. Overexpression of KLC2 Due to a Homozygous Deletion in the Non-Coding Region Causes SPOAN Syndrome. *Human Molecular Genetics* 24 (24): 6877–85. https://doi.org/10.1093/hmg/ddv388.

Mendoza-Ferreira, Natalia, Marie Coutelier, Eva Janzen, Seyyedmohsen Hosseinibarkooie, Heiko Löhr, Svenja Schneider, et al. 2018. Biallelic CHP1 Mutation Causes Human Autosomal Recessive Ataxia by Impairing NHE1 Function. *Neurology: Genetics* 4 (1). https://doi.org/10.1212/NXG.0000000000000209.

Minnerop, Martina, Delia Kurzwelly, Holger Wagner, Anne S. Soehn, Jennifer Reichbauer, Feifei Tao, et al. 2017. Hypomorphic Mutations in POLR3A Are a Frequent Cause of Sporadic and Recessive Spastic Ataxia. *Brain* 140 (6): 1561–78. https://doi.org/10.1093/brain/awx095.

Mollet, Julie, Agnès Delahodde, Valérie Serre, Dominique Chretien, Dimitri Schlemmer, Anne Lombes, et al. 2008. CABC1 Gene Mutations Cause Ubiquinone Deficiency with Cerebellar Ataxia and Seizures. *American Journal of Human Genetics* 82 (3): 623–30. https://doi.org/10.1016/j.ajhg.2007.12.022.

Montenegro, Gladys, Adriana P. Rebelo, James Connell, Rachel Allison, Carla Babalini, Michela D'Aloia, et al. 2012. Mutations in the ER-Shaping Protein Reticulon 2 Cause the Axon-Degenerative Disorder Hereditary Spastic Paraplegia Type 12. *Journal of Clinical Investigation* 122 (2): 538–44. https://doi.org/10.1172/JCI60560.

Moreira, Maria Céu, Sandra Klur, Mitsunori Watanabe, Andrea H. Németh, Isabelle Le Ber, José Carlos Moniz, et al. 2004. Senataxin, the Ortholog of a Yeast RNA Helicase, Is Mutant in Ataxia-Ocular Apraxia 2. *Nature Genetics* 36 (3): 225–27. https://doi.org/10.1038/ng1303.

Moreno-De-Luca, Andres, Sandra L. Helmers, Hui Mao, Thomas G. Burns, Amanda M.A. Melton, Karen R. Schmidt, et al. 2011. Adaptor Protein Complex-4 (AP-4) Deficiency Causes a Novel Autosomal Recessive Cerebral Palsy Syndrome with Microcephaly and Intellectual Disability. *Journal of Medical Genetics* 48 (2): 141–44. https://doi.org/10.1136/jmg.2010.082263.

Moseley, Melinda L., Tao Zu, Yoshio Ikeda, Wangcai Gao, Anne K. Mosemiller, Randy S. Daughters, et al. 2006. Bidirectional Expression of CUG and CAG Expansion Transcripts and Intranuclear Polyglutamine Inclusions in Spinocerebellar Ataxia Type 8. *Nature*

Genetics 38 (7): 758–69. https://doi.org/10.1038/ng1827.

Nibbeling, Esther A.R., Anna Duarri, Corien C. Verschuuren-Bemelmans, Michiel R. Fokkens, Juha M. Karjalainen, Cleo J.L.M. Smeets, et al. 2017. Exome Sequencing and Network Analysis Identifies Shared Mechanisms Underlying Spinocerebellar Ataxia. *Brain* 140 (11): 2860–78. https://doi.org/10.1093/brain/awx251.

Novarino, Gaia, Ali G. Fenstermaker, Maha S. Zaki, Matan Hofree, Jennifer L. Silhavy, Andrew D. Heiberg, et al. 2014. Exome Sequencing Links Corticospinal Motor Neuron Disease to Common Neurodegenerative Disorders. *Science* 343 (6170): 506–11. https://doi.org/10.1126/science.1247363.

Oates, Emily C., Alexander M. Rossor, Majid Hafezparast, Michael Gonzalez, Fiorella Speziani, Daniel G. Macarthur, et al. 2013. Mutations in BICD2 Cause Dominant Congenital Spinal Muscular Atrophy and Hereditary Spastic Paraplegia. *American Journal of Human Genetics* 92 (6): 965–73. https://doi.org/10.1016/j.ajhg.2013.04.018.

Onat, Onur Emre, Suleyman Gulsuner, Kaya Bilguvar, Ayse Nazli Basak, Haluk Topaloglu, Meliha Tan, et al. 2013. Missense Mutation in the ATPase, Aminophospholipid Transporter Protein ATP8A2 Is Associated with Cerebellar Atrophy and Quadrupedal Locomotion. *European Journal of Human Genetics* 21 (3): 281–85. https://doi.org/10.1038/ejhg.2012.170.

Ophoff, Roel A., Gisela M. Terwindt, Monique N. Vergouwe, Ronald Van Eijk, Peter J. Oefner, Susan M.G. Hoffman, et al. 1996. Familial Hemiplegic Migraine and Episodic Ataxia Type-2 Are Caused by Mutations in the Ca2+ Channel Gene CACNL1A4. *Cell* 87 (3): 543–52. https://doi.org/10.1016/S0092-8674(00)81373-2.

Orlacchio, A., C. Patrono, F. Gaudiello, C. Rocchi, V. Moschella, R. Floris, et al. 2008. Silver Syndrome Variant of Hereditary Spastic Paraplegia: A Locus to 4p and Allelism with SPG4. *Neurology* 70 (21): 1959–66. https://doi.org/10.1212/01.wnl.0000294330.27058.61.

Orlacchio, Antonio, Toshitaka Kawarai, Fabrizio Gaudiello, Peter H. St. George-Hyslop, Roberto Floris, and Giorgio Bernardi. 2005. New Locus for Hereditary Spastic Paraplegia Maps to Chromosome 1p31.1-1p21.1. *Annals of Neurology* 58 (3): 423–29. https://doi.org/10.1002/ana.20590.

Orthmann-Murphy, Jennifer L., Ettore Salsano, Charles K. Abrams, Alberto Bizzi, Graziella Uziel, Mona M. Freidin, et al. 2009. Hereditary Spastic Paraplegia Is a Novel Phenotype for GJA12/GJC2 Mutations. *Brain* 132 (2): 426–38. https://doi.org/10.1093/brain/awn328.

Ouahchi, Karim, Makoto Arita, Herbert Kayden, Fayçal Hentati, Mongi Ben Hamida, Ronald Sokol, et al. 1995. Ataxia with Isolated Vitamin E Deficiency Is Caused by Mutations in the α–Tocopherol Transfer Protein. *Nature Genetics* 9 (2): 141–45. https://doi.org/10.1038/ng0295-141.

Oz-Levi, Danit, Bruria Ben-Zeev, Elizabeth K. Ruzzo, Yuki Hitomi, Amir Gelman, Kimberly Pelak, et al. 2012. Mutation in TECPR2 Reveals a Role for Autophagy in Hereditary Spastic Paraparesis. *American Journal of Human Genetics* 91 (6): 1065–72.

https://doi.org/10.1016/j.ajhg.2012.09.015.

Ozes, B., N. Karagoz, R. Schüle, A. Rebelo, M. J. Sobrido, F. Harmuth, et al. 2017. PLA2G6 Mutations Associated with a Continuous Clinical Spectrum from Neuroaxonal Dystrophy to Hereditary Spastic Paraplegia. *Clinical Genetics* 92 (5): 534–39. https://doi.org/10.1111/cge.13008.

Patel, Heema, Harold Cross, Christos Proukakis, Ruth Hershberger, Peer Bork, Francesca D. Ciccarelli, et al. 2002. SPG20 Is Mutated in Troyer Syndrome, an Hereditary Spastic Paraplegia. *Nature Genetics* 31 (4): 347–48. https://doi.org/10.1038/ng937.

Pierson, Tyler Mark, David Adams, Florian Bonn, Paola Martinelli, Praveen F. Cherukuri, Jamie K. Teer, et al. 2011. Whole-Exome Sequencing Identifies Homozygous AFG3L2 Mutations in a Spastic Ataxia-Neuropathy Syndrome Linked to Mitochondrial m-AAA Proteases. *PLoS Genetics* 7 (10). https://doi.org/10.1371/journal.pgen.1002325.

Pulst, Stefan M., Alex Nechiporuk, Tamilla Nechiporuk, Suzana Gispert, Xiao Ning Chen, Iscia Lopes-Cendes, et al. 1996. Moderate Expansion of a Normally Biallelic Trinucleotide Repeat in Spinooerebellar Ataxia Type. *Nature Genetics* 14 (3): 269–76. https://doi.org/10.1038/ng1196-269.

Rainier, Shirley, Melanie Bui, Erin Mark, Donald Thomas, Debra Tokarz, Lei Ming, et al. 2008. Neuropathy Target Esterase Gene Mutations Cause Motor Neuron Disease. *American Journal of Human Genetics* 82 (3): 780–85. https://doi.org/10.1016/j.ajhg.2007.12.018.

Rainier, Shirley, Jing Hua Chai, Debra Tokarz, Robert D. Nicholls, and John K. Fink. 2003. NIPA1 Gene Mutations Cause Autosomal Dominant Hereditary Spastic Paraplegia (SPG6). *American Journal of Human Genetics* 73 (4): 967–71. https://doi.org/10.1086/378817.

Reid, Evan, Mark Kloos, Allison Ashley-Koch, Lori Hughes, Simon Bevan, Ingrid K. Svenson, et al. 2002. A Kinesin Heavy Chain (KIF5A) Mutation in Hereditary Spastic Paraplegia (SPG10). *American Journal of Human Genetics* 71 (5): 1189–94. https://doi.org/10.1086/344210.

Rinaldi, Carlo, Thomas Schmidt, Alan J. Situ, Janel O. Johnson, Philip R. Lee, Ke Lian Chen, et al. 2015. Mutation in CPT1C Associated with Pure Autosomal Dominant Spastic Paraplegia. *JAMA Neurology* 72 (5): 561–70. https://doi.org/10.1001/jamaneurol.2014.4769.

Rydning, S. L., A. Dudesek, F. Rimmele, C. Funke, S. Krüger, S. Biskup, et al. 2018. A Novel Heterozygous Variant in ERLIN2 Causes Autosomal Dominant Pure Hereditary Spastic Paraplegia. *European Journal of Neurology* 25 (7): 943–48. https://doi.org/10.1111/ene.13625.

Sagnelli, Anna, Stefania Magri, Laura Farina, Luisa Chiapparini, Giorgio Marotta, Davide Tonduti, et al. 2016. Early-Onset Progressive Spastic Paraplegia Caused by a Novel TUBB4A Mutation: Brain MRI and FDG-PET Findings. *Journal of Neurology*. Dr. Dietrich Steinkopff Verlag GmbH and Co. KG. https://doi.org/10.1007/s00415-016-8020-8.

Sánchez-Ferrero, E., E. Coto, C. Beetz, J. Gámez, Ai Corao, M. Díaz, et al. 2013. SPG7 Mutational Screening in Spastic Paraplegia Patients Supports a Dominant Effect for Some

Mutations and a Pathogenic Role for p.A510V. *Clinical Genetics* 83 (3): 257–62. https://doi.org/10.1111/j.1399-0004.2012.01896.x.

Sarzi, Emmanuelle, Steffi Goffart, Valérie Serre, Dominique Chrétien, Abdelhamid Slama, Arnold Munnich, et al. 2007. Twinkle Helicase (PEO1) Gene Mutation Causes Mitochondrial DNA Depletion. *Annals of Neurology* 62 (6): 579–87. https://doi.org/10.1002/ana.21207.

Sato, Nozomu, Takeshi Amino, Kazuhiro Kobayashi, Shuichi Asakawa, Taro Ishiguro, Taiji Tsunemi, et al. 2009. Spinocerebellar Ataxia Type 31 Is Associated with “Inserted” Penta-Nucleotide Repeats Containing (TGGAA)N. *American Journal of Human Genetics* 85 (5): 544–57. https://doi.org/10.1016/j.ajhg.2009.09.019.

Saugier-Veber, Pascale, Arnold Munnich, Dominique Bonneau, Jean Michel Rozet, Martine le Merrer, Roger Gil, et al. 1994. X-Linked Spastic Paraplegia and Pelizaeus-Merzbacher Disease Are Allelic Disorders at the Proteolipid Protein Locus. *Nature Genetics* 6 (3): 257–62. https://doi.org/10.1038/ng0394-257.

Savitsky, Kinneret, Anat Bar-Shira, Shlomit Gilad, Galit Rotman, Yael Ziv, Lina Vanagaite, et al. 1995. A Single Ataxia Telangiectasia Gene with a Product Similar to PI-3 Kinase. *Science* 268 (5218): 1749–53. https://doi.org/10.1126/science.7792600.

Schmidt, Wolfgang M., S. Lane Rutledge, Rebecca Schüle, Benjamin Mayerhofer, Stephan Züchner, Eugen Boltshauser, et al. 2015. Disruptive SCYL1 Mutations Underlie a Syndrome Characterized by Recurrent Episodes of Liver Failure, Peripheral Neuropathy, Cerebellar Atrophy, and Ataxia. *American Journal of Human Genetics* 97 (6): 855–61. https://doi.org/10.1016/j.ajhg.2015.10.011.

Scholl, Ute I., Murim Choi, Tiewen Liu, Vincent T. Ramaekers, Martin G. Häusler, Joanne Grimmer, et al. 2009. Seizures, Sensorineural Deafness, Ataxia, Mental Retardation, and Electrolyte Imbalance (SeSAME Syndrome) Caused by Mutations in KCNJ10. *Proceedings of the National Academy of Sciences of the United States of America* 106 (14): 5842–47. https://doi.org/10.1073/pnas.0901749106.

Schüle, R., M. Bonin, A. Dürr, S. Forlani, A. D. Sperfeld, S. Klimpe, et al. 2009. Autosomal Dominant Spastic Paraplegia with Peripheral Neuropathy Maps to Chr12q23-24. *Neurology* 72 (22): 1893–98. https://doi.org/10.1212/WNL.0b013e3181a6086c.

Schuurs-Hoeijmakers, Janneke H.M., Michael T. Geraghty, Erik Jan Kamsteeg, Salma Ben-Salem, Susanne T. De Bot, Bonnie Nijhof, et al. 2012. Mutations in DDHD2, Encoding an Intracellular Phospholipase A1, Cause a Recessive Form of Complex Hereditary Spastic Paraplegia. *American Journal of Human Genetics* 91 (6): 1073–81. https://doi.org/10.1016/j.ajhg.2012.10.017.

Seixas, Ana I., Joana R. Loureiro, Cristina Costa, Andrés Ordóñez-Ugalde, Hugo Marcelino, Cláudia L. Oliveira, et al. 2017. A Pentanucleotide ATTTC Repeat Insertion in the Non-Coding Region of DAB1, Mapping to SCA37, Causes Spinocerebellar Ataxia. *American Journal of Human Genetics* 101 (1): 87–103. https://doi.org/10.1016/j.ajhg.2017.06.007.

Sellick, Gabrielle S., Karen T. Barker, Irene Stolte-Dijkstra, Christina Fleischmann, Richard J. Coleman, Christine Garrett, et al. 2004. Mutations in PTF1A Cause Pancreatic and Cerebellar Agenesis. *Nature Genetics* 36 (12): 1301–5. https://doi.org/10.1038/ng1475.

Seong, Eunju, Ryan Insolera, Marija Dulovic, Erik Jan Kamsteeg, Joanne Trinh, Norbert Brüggemann, et al. 2018. Mutations in VPS13D Lead to a New Recessive Ataxia with Spasticity and Mitochondrial Defects. *Annals of Neurology* 83 (6): 1075–88. https://doi.org/10.1002/ana.25220.

Shi, Yuting, Junling Wang, Jia Da Li, Haigang Ren, Wenjuan Guan, Miao He, et al. 2013. Identification of CHIP as a Novel Causative Gene for Autosomal Recessive Cerebellar Ataxia. *PLoS ONE* 8 (12). https://doi.org/10.1371/journal.pone.0081884.

Shimazaki, Haruo, Junko Honda, Tametou Naoi, Michito Namekawa, Imaharu Nakano, Masahide Yazaki, et al. 2014. Autosomal-Recessive Complicated Spastic Paraplegia with a Novel Lysosomal Trafficking Regulator Gene Mutation. *Journal of Neurology, Neurosurgery and Psychiatry* 85 (9): 1024–28. https://doi.org/10.1136/jnnp-2013-306981.

Shimazaki, Haruo, Yoshihisa Takiyama, Hiroyuki Ishiura, Chika Sakai, Yuichi Matsushima, Hideyuki Hatakeyama, et al. 2012. A Homozygous Mutation of C12orf65 Causes Spastic Paraplegia with Optic Atrophy and Neuropathy (SPG55). *Journal of Medical Genetics* 49 (12): 777–84. https://doi.org/10.1136/jmedgenet-2012-101212.

Simpson, Michael A., Harold Cross, Christos Proukakis, Anna Pryde, Ruth Hershberger, Arnaud Chatonnet, et al. 2003. Maspardin Is Mutated in Mast Syndrome, a Complicated Form of Hereditary Spastic Paraplegia Associated with Dementia. *American Journal of Human Genetics* 73 (5): 1147–56. https://doi.org/10.1086/379522.

Słabicki, Mikołaj, Mirko Theis, Dragomir B. Krastev, Sergey Samsonov, Emeline Mundwiller, Magno Junqueira, et al. 2010. A Genome-Scale DNA Repair RNAi Screen Identifies SPG48 as a Novel Gene Associated with Hereditary Spastic Paraplegia. *PLoS Biology* 8 (6). https://doi.org/10.1371/journal.pbio.1000408.

Spiegel, Ronen, Ophry Pines, Asaf Ta-Shma, Efrat Burak, Avraham Shaag, Jonatan Halvardson, et al. 2012. Infantile Cerebellar-Retinal Degeneration Associated with a Mutation in Mitochondrial Aconitase, ACO2. *American Journal of Human Genetics* 90 (3): 518–23. https://doi.org/10.1016/j.ajhg.2012.01.009.

Steckley, J. L., George C. Ebers, M. Z. Cader, and R. S. McLachlan. 2001. An Autosomal Dominant Disorder with Episodic Ataxia, Vertigo, and Tinnitus. *Neurology* 57 (8): 1499–1502. https://doi.org/10.1212/WNL.57.8.1499.

Steinmuller, R., A. Lantigua-Cruz, R. Garcia-Garcia, M. Kostrzewa, D. Steinberger, and U. Muller. 1997. Evidence of a Third Locus in X-Linked Recessive Spastic Paraplegia [1]. *Human Genetics*. Hum Genet. https://doi.org/10.1007/s004390050507.

Stevanin, G., C. Paternotte, P. Coutinho, S. Klebe, N. Elleuch, J. L. Loureiro, et al. 2007. A New Locus for Autosomal Recessive Spastic Paraplegia (SPG32) on Chromosome 14q12-Q21. *Neurology* 68 (21): 1837–40. https://doi.org/10.1212/01.wnl.0000262043.53386.22.

Stevanin, Giovanni, Naima Bouslam, Stéphane Thobois, Hamid Azzedine, Lucas Ravaux, Anne Boland, et al. 2004. Spinocerebellar Ataxia with Sensory Neuropathy (SCA25) Maps to Chromosome 2p. *Annals of Neurology* 55 (1): 97–104. https://doi.org/10.1002/ana.10798.

Stevanin, Giovanni, Filippo M. Santorelli, Hamid Azzedine, Paula Coutinho, Jacques Chomilier, Paola S. Denora, et al. 2007. Mutations in SPG11, Encoding Spatacsin, Are a Major Cause of Spastic Paraplegia with Thin Corpus Callosum. *Nature Genetics* 39 (3): 366–72. https://doi.org/10.1038/ng1980.

Stewart, Grant S., Richard S. Maser, Tanja Stankovic, Debra A. Bressan, Mark I. Kaplan, Nikolaas G.J. Jaspers, et al. 1999. The DNA Double-Strand Break Repair Gene HMRE11 Is Mutated in Individuals with an Ataxia-Telangiectasia-like Disorder. *Cell* 99 (6): 577–87. https://doi.org/10.1016/S0092-8674(00)81547-0.

Storey, E., M. Bahlo, M. Fahey, O. Sisson, C. J. Lueck, and R. J.M. Gardner. 2009. A New Dominantly Inherited Pure Cerebellar Ataxia, SCA 30. *Journal of Neurology, Neurosurgery and Psychiatry* 80 (4): 408–11. https://doi.org/10.1136/jnnp.2008.159459.

Sun, Yu, Rowida Almomani, Guido J. Breedveld, Gijs W.E. Santen, Emmelien Aten, Dirk J. Lefeber, et al. 2013. Autosomal Recessive Spinocerebellar Ataxia 7 (SCAR7) Is Caused by Variants in TPP1, The Gene Involved in Classic Late-Infantile Neuronal Ceroid Lipofuscinosis 2 Disease (CLN2 Disease). *Human Mutation* 34 (5): 706–13. https://doi.org/10.1002/humu.22292.

Swieten, John C. Van, Esther Brusse, Bianca M. De Graaf, Elmar Krieger, Raoul Van de Graaf, Inge De Koning, et al. 2003. A Mutation in the Fibroblast Growth Factor 14 Gene Is Associated with Autosomal Dominant Cerebral Ataxia. *American Journal of Human Genetics* 72 (1): 191–99. https://doi.org/10.1086/345488.

Synofzik, Matthis, Michael A. Gonzalez, Charles Marques Lourenco, Marie Coutelier, Tobias B. Haack, Adriana Rebelo, et al. 2014. PNPLA6 Mutations Cause Boucher-Neuhäuser and Gordon Holmes Syndromes as Part of a Broad Neurodegenerative Spectrum. *Brain* 137 (1): 69–77. https://doi.org/10.1093/brain/awt326.

Takashima, Hiroshi, Cornelius F. Boerkoel, Joy John, Gulam Mustafa Saifi, Mustafa A.M. Salih, Dawna Armstrong, et al. 2002. Mutation of TDP1, Encoding a Topoisomerase I-Dependent DNA Damage Repair Enzyme, in Spinocerebellar Ataxia with Axonal Neuropathy. *Nature Genetics* 32 (2): 267–72. https://doi.org/10.1038/ng987.

Tassan, Nada Al, Dania Khalil, Jameela Shinwari, Latifa Al Sharif, Prashant Bavi, Zainularifeen Abduljaleel, et al. 2012. A Missense Mutation in PIK3R5 Gene in a Family with Ataxia and Oculomotor Apraxia. *Human Mutation* 33 (2): 351–54. https://doi.org/10.1002/humu.21650.

Tesson, Christelle, Magdalena Nawara, Mustafa A.M. Salih, Rodrigue Rossignol, Maha S. Zaki, Mohammed Al Balwi, et al. 2012. Alteration of Fatty-Acid-Metabolizing Enzymes Affects Mitochondrial Form and Function in Hereditary Spastic Paraplegia. *American Journal of Human Genetics* 91 (6): 1051–64. https://doi.org/10.1016/j.ajhg.2012.11.001.

Thomas, Anna C., Hywel Williams, Núria Setõ-Salvia, Chiara Bacchelli, Dagan Jenkins, Mary

O’Sullivan, et al. 2014. Mutations in SNX14 Cause a Distinctive Autosomal-Recessive Cerebellar Ataxia and Intellectual Disability Syndrome. *American Journal of Human Genetics* 95 (5): 611–21. https://doi.org/10.1016/j.ajhg.2014.10.007.

Tranebjaerg, Lisbeth, Tanya M. Teslovich, Mary Pat Jones, M. Michael Barmada, Toril Fagerheim, Arve Dahl, et al. 2003. Genome-Wide Homozygosity Mapping Localizes a Gene for Autosomal Recessive Non-Progressive Infantile Ataxia to 20q11-Q13. *Human Genetics* 113 (3): 293–95. https://doi.org/10.1007/s00439-003-0967-8.

Troelstra, Christine, Alain van Gool, Jan de Wit, Wim Vermeulen, Dirk Bootsma, and Jan H.J. Hoeijmakers. 1992. ERCC6, a Member of a Subfamily of Putative Helicases, Is Involved in Cockayne’s Syndrome and Preferential Repair of Active Genes. *Cell* 71 (6): 939–53. https://doi.org/10.1016/0092-8674(92)90390-X.

Tsaousidou, Maria K., Karim Ouahchi, Tom T. Warner, Yi Yang, Michael A. Simpson, Nigel G G. Laing, et al. 2008. Sequence Alterations within CYP7B1 Implicate Defective Cholesterol Homeostasis in Motor-Neuron Degeneration. *American Journal of Human Genetics* 82 (2): 510–15. https://doi.org/10.1016/j.ajhg.2007.10.001.

Tsoi, Ho, Allen C.S. Yu, Zhefan S. Chen, Nelson K.N. Ng, Anne Y.Y. Chan, Liz Y.P. Yuen, et al. 2014. A Novel Missense Mutation in CCDC88C Activates the JNK Pathway and Causes a Dominant Form of Spinocerebellar Ataxia. *Journal of Medical Genetics* 51 (9): 590–95. https://doi.org/10.1136/jmedgenet-2014-102333.

Utine, G. Eda, Göknur Haliloğlu, Bilge Salanci, Arda Çetinkaya, P. Özlem Kiper, Yasemin Alanay, et al. 2013. A Homozygous Deletion in GRID2 Causes a Human Phenotype with Cerebellar Ataxia and Atrophy. *Journal of Child Neurology* 28 (7): 926–32. https://doi.org/10.1177/0883073813484967.

Valdmanis, Paul N., Inge A. Meijer, Annie Reynolds, Adrienne Lei, Patrick MacLeod, David Schlesinger, et al. 2007. Mutations in the KIAA0196 Gene at the SPG8 Locus Cause Hereditary Spastic Paraplegia. *American Journal of Human Genetics* 80 (1): 152–61. https://doi.org/10.1086/510782.

Varon, Raymonda, Christine Vissinga, Matthias Platzer, Karen M. Cerosaletti, Krystyna H. Chrzanowska, Kathrin Saar, et al. 1998. Nibrin, a Novel DNA Double-Strand Break Repair Protein, Is Mutated in Nijmegen Breakage Syndrome. *Cell* 93 (3): 467–76. https://doi.org/10.1016/S0092-8674(00)81174-5.

Vaz, Frédéric M., John H. McDermott, Mariëlle Alders, Saskia B. Wortmann, Stefan Kölker, Mia L. Pras-Raves, et al. 2019. Mutations in PCYT2 Disrupt Etherlipid Biosynthesis and Cause a Complex Hereditary Spastic Paraplegia. *Brain* 142 (11): 3382–97. https://doi.org/10.1093/brain/awz291.

Vazza, G., M. Zortea, F. Boaretto, G. F. Micaglio, V. Sartori, and M. L. Mostacciuolo. 2000. A New Locus for Autosomal Recessive Spastic Paraplegia Associated with Mental Retardation and Distal Motor Neuropathy, SPG14, Maps to Chromosome 3q27-Q28. *American Journal of Human Genetics* 67 (2): 504–9. https://doi.org/10.1086/303017.

Verkerk, Annemieke J.M.H., Rachel Schot, Belinda Dumee, Karlijn Schellekens, Sigrid Swagemakers, Aida M. Bertoli-Avella, et al. 2009. Mutation in the AP4M1 Gene Provides a Model for Neuroaxonal Injury in Cerebral Palsy. *American Journal of Human Genetics* 85 (1): 40–52. https://doi.org/10.1016/j.ajhg.2009.06.004.

Vermeer, Sascha, Alexander Hoischen, Rowdy P.P. Meijer, Christian Gilissen, Kornelia Neveling, Nienke Wieskamp, et al. 2010. Targeted Next-Generation Sequencing of a 12.5 Mb Homozygous Region Reveals ANO10 Mutations in Patients with Autosomal-Recessive Cerebellar Ataxia. *American Journal of Human Genetics* 87 (6): 813–19. https://doi.org/10.1016/j.ajhg.2010.10.015.

Verny, Christophe, Naig Guegen, Valerie Desquiret, Arnaud Chevrollier, Adriana Prundean, Frederic Dubas, et al. 2011. Hereditary Spastic Paraplegia-like Disorder Due to a Mitochondrial ATP6 Gene Point Mutation. *Mitochondrion* 11 (1): 70–75. https://doi.org/10.1016/j.mito.2010.07.006.

Waalkens, Anne J.E., Fleur Vansenne, Annemarie H. Van Der Hout, Rodi Zutt, Jeroen Mourmans, Eduardo Tolosa, et al. 2018. Expanding the ADCY5 Phenotype toward Spastic Paraparesis: Amutation in the M2 Domain. *Neurology: Genetics* 4 (1). https://doi.org/10.1212/NXG.0000000000000214.

Wagner, Matias, Daniel P.S. Osborn, Ina Gehweiler, Maike Nagel, Ulrike Ulmer, Somayeh Bakhtiari, et al. 2019. Bi-Allelic Variants in RNF170 Are Associated with Hereditary Spastic Paraplegia. *Nature Communications* 10 (1). https://doi.org/10.1038/s41467-019-12620-9.

Wang, Jun, Jun Ling Wang, Xu Yang, Kun Xia, Zheng Mao Hu, Ling Weng, et al. 2010. TGM6 Identified as a Novel Causative Gene of Spinocerebellar Ataxias Using Exome Sequencing. *Brain* 133 (12): 3510–18. https://doi.org/10.1093/brain/awq323.

Warrenburg, Bart P. Van De, Meyke I. Schouten, Susanne T. De Bot, Sascha Vermeer, Rowdy Meijer, Maartje Pennings, et al. 2016. Clinical Exome Sequencing for Cerebellar Ataxia and Spastic Paraplegia Uncovers Novel Gene-Disease Associations and Unanticipated Rare Disorders. *European Journal of Human Genetics* 24 (10): 1460–66. https://doi.org/10.1038/ejhg.2016.42.

Waters, Michael F., Ngatali A. Minassian, Giovanni Stevanin, Karla P. Figueroa, John P.A. Bannister, Dagmar Nolte, et al. 2006. Mutations in Voltage-Gated Potassium Channel KCNC3 Cause Degenerative and Developmental Central Nervous System Phenotypes. *Nature Genetics* 38 (4): 447–51. https://doi.org/10.1038/ng1758.

Watson, Lauren M., Elizabeth Bamber, Ricardo Parolin Schnekenberg, Jonathan Williams, Conceição Bettencourt, Jennifer Lickiss, et al. 2017. Dominant Mutations in GRM1 Cause Spinocerebellar Ataxia Type 44. *American Journal of Human Genetics* 101 (3): 451–58. https://doi.org/10.1016/j.ajhg.2017.08.005.

Windpassinger, Christian, Michaela Auer-Grumbach, Joy Irobi, Heema Patel, Erwin Petek, Gerd Hörl, et al. 2004. Heterozygous Missense Mutations in BSCL2 Are Associated with Distal Hereditary Motor Neuropathy and Silver Syndrome. *Nature Genetics* 36 (3): 271–76.

https://doi.org/10.1038/ng1313.

Winkelmann, Juliane, Ling Lin, Barbara Schormair, Birgitte R. Kornum, Juliette Faraco, Giuseppe Plazzi, et al. 2012. Mutations in DNMT1 Cause Autosomal Dominant Cerebellar Ataxia, Deafness and Narcolepsy. *Human Molecular Genetics* 21 (10): 2205–10. https://doi.org/10.1093/hmg/dds035.

Winterthun, S., G. Ferrari, L. He, R. W. Taylor, M. Zeviani, D. M. Turnbull, et al. 2005. Autosomal Recessive Mitochondrial Ataxic Syndrome Due to Mitochondrial Polymerase γ Mutations. *Neurology* 64 (7): 1204–8. https://doi.org/10.1212/01.WNL.0000156516.77696.5A.

Yang, Ying, Wei Liu, Zhipeng Fang, Juan Shi, Fengyu Che, Chunxia He, et al. 2016. A Newly Identified Missense Mutation in FARS2 Causes Autosomal-Recessive Spastic Paraplegia. *Human Mutation* 37 (2): 165–69. https://doi.org/10.1002/humu.22930.

Yildirim, Yeşerin, Elif Kocasoy Orhan, Sibel Aylin Ugur Iseri, Piraye Serdaroglu-Oflazer, Bülent Kara, Seyhun Solakoğlu, et al. 2011. A Frameshift Mutation of ERLIN2 in Recessive Intellectual Disability, Motor Dysfunction and Multiple Joint Contractures. *Human Molecular Genetics* 20 (10): 1886–92. https://doi.org/10.1093/hmg/ddr070.

Ylikallio, Emil, Doyoun Kim, Pirjo Isohanni, Mari Auranen, Eunjoon Kim, Tuula Lönnqvist, et al. 2015. Dominant Transmission of de Novo KIF1A Motor Domain Variant Underlying Pure Spastic Paraplegia. *European Journal of Human Genetics* 23 (10): 1427–30. https://doi.org/10.1038/ejhg.2014.297.

Zanni, Ginevra, Tito Calì, Vera M. Kalscheuer, Denis Ottolini, Sabina Barresi, Nicolas Lebrun, et al. 2012. Mutation of Plasma Membrane Ca2+ ATPase Isoform 3 in a Family with X-Linked Congenital Cerebellar Ataxia Impairs Ca2+ Homeostasis. *Proceedings of the National Academy of Sciences of the United States of America* 109 (36): 14514–19. https://doi.org/10.1073/pnas.1207488109.

Zhao, Guo Hua, Zheng Mao Hu, Lu Shen, Hong Jiang, Zhi Jun Ren, Xiao Min Liu, et al. 2008. A Novel Candidate Locus on Chromosome 11p14.1-P11.2 for Autosomal Dominant Hereditary Spastic Paraplegia. *Chinese Medical Journal* 121 (5): 430–34. https://doi.org/10.1097/00029330-200803010-00012.

Zhao, Xinping, David Alvarado, Shirley Rainier, Rosemary Lemons, Peter Hedera, Christian H. Weber, et al. 2001. Mutations in a Newly Identified GTPase Gene Cause Autosomal Dominant Hereditary Spastic Paraplegia. *Nature Genetics* 29 (3): 326–31. https://doi.org/10.1038/ng758.

Zhuchenko, Olga, Jennifer Bailey, Penelope Bonnen, Tetsuo Ashizawa, David W. Stockton, Chris Amos, et al. 1997. Autosomal Dominant Cerebellar Ataxia (SCA6) Associated with Small Polyglutamine Expansions in the α(1A)-Voltage-Dependent Calcium Channel. *Nature Genetics*. Nat Genet. https://doi.org/10.1038/ng0197-62.

Zivony-Elboum, Yifat, Wendy Westbroek, Nehama Kfir, David Savitzki, Yishay Shoval, Assnat Bloom, et al. 2012. A Founder Mutation in Vps37A Causes Autosomal Recessive Complex

Hereditary Spastic Paraparesis. *Journal of Medical Genetics* 49 (7): 462–72. https://doi.org/10.1136/jmedgenet-2012-100742.

Zortea, M., A. Vettori, C. P. Trevisan, S. Bellini, G. Vazza, M. Armani, et al. 2002. Genetic Mapping of a Susceptibility Locus for Disc Herniation and Spastic Paraplegia on 6q23.3-Q24.1. *Journal of Medical Genetics* 39 (6): 387–90. https://doi.org/10.1136/jmg.39.6.387.

Züchner, Stephan, Gaofeng Wang, Khanh Nhat Tran-Viet, Martha A. Nance, Perry C. Gaskell, Jeffery M. Vance, et al. 2006. Mutations in the Novel Mitochondrial Protein REEP1 Cause Hereditary Spastic Paraplegia Type 31. *American Journal of Human Genetics* 79 (2): 365–69. https://doi.org/10.1086/505361.
